# Comprehensive decoding mental processes from Web repositories of functional brain images

**DOI:** 10.1038/s41598-022-10710-1

**Published:** 2022-04-29

**Authors:** Romuald Menuet, Raphael Meudec, Jérôme Dockès, Gael Varoquaux, Bertrand Thirion

**Affiliations:** 1 Parietal, Inria, Saclay, France; 2 Neurospin, CEA, Saclay, France; 3 Université Paris-Saclay, Saclay, France; 4 McGill University, Montreal, Canada; 5 Owkin Lab, Paris, France

**Keywords:** Neural decoding, Neural encoding

## Abstract

Associating brain systems with mental processes requires statistical analysis of brain activity across many cognitive processes. These analyses typically face a difficult compromise between scope—from domain-specific to system-level analysis—and accuracy. Using all the functional Magnetic Resonance Imaging (fMRI) statistical maps of the largest data repository available, we trained machine-learning models that decode the cognitive concepts probed in unseen studies. For this, we leveraged two comprehensive resources: NeuroVault—an open repository of fMRI statistical maps with unconstrained annotations—and Cognitive Atlas—an ontology of cognition. We labeled NeuroVault images with Cognitive Atlas concepts occurring in their associated metadata. We trained neural networks to predict these cognitive labels on tens of thousands of brain images. Overcoming the heterogeneity, imbalance and noise in the training data, we successfully decoded more than 50 classes of mental processes on a large test set. This success demonstrates that image-based meta-analyses can be undertaken at scale and with minimal manual data curation. It enables broad reverse inferences, that is, concluding on mental processes given the observed brain activity.

## Introduction

Cognitive neuroscience probes the relationships between mental functions and brain systems by contrasting brain responses observed in conditions that involve these functions to control conditions. For instance, in task functional Magnetic Resonance Imaging (fMRI) studies, experimental protocols trigger cognitive processes in a set of participants, while the corresponding neural activity is recorded. Standard statistical analysis is based on a subtractive logic: fMRI data are used to measure local differences in brain oxygen supply between two experimental conditions, a *target* condition that includes the cognitive *concept* of interest^1^ and a *control* condition that is either a related task or a baseline^2^.

To characterize the function of the recruited brain structures, it is now common to input these data to machine learning models, to infer mental processes from brain activity recordings^3^. Such *decoding* procedures are used in neuroscience to detect brain activation patterns that characterize the underlying mental processes. Decoding complements standard cognitive images analyses, that detect brain responses related to behavior—an approach called *encoding*^4^. While standard encoding analyses inform on the brain activity under a given manipulation, they do not give evidence that the activation of a given area is specific to the mental process under consideration^5^. There is hope that decoding can ground *reverse inference*, i.e. drawing conclusion on the function of a brain structure^6,7^.

Decoding is usually performed at the subject or study level, on a limited set of mutually exclusive concepts, in which case it does not support reverse inference across cognitive domains. By contrast, meta-analyses leverage several studies, but their scope is typically restricted to a given cognitive domain, and when performed at a large scale, they usually consider encoding-type analysis, so that it remains unclear whether reverse inference is possible. Decoding in the context of Image-Based Meta-Analyses (IBMA) has been shown to achieve good performance^7,8^, but only in restricted sets of studies, discriminating between few cognitive concepts. This has limited the ability of IBMA to assess the selectivity of a region activation, and, as a consequence, the validity of corresponding reverse inferences^9^. Automated Coordinate-Based Meta-Analyses (CBMA) use many more studies. In recent works^10–12^, CBMA has been used to map a large set of concepts by using stereotactic coordinate tables from articles, but the underlying data representation loses a lot of information, leading to sub-optimal performance.

In computer vision, aggregating huge heterogeneous datasets from the Internet, as ImageNet^13^, has proved to be crucial to solve automated image understanding. Similar undertakings in neuroimaging could yield a more exhaustive functional mapping of the brain. Indeed, heterogeneity can be a strength, as it grounds broader generalization^14,15^. In this study, we expand the scope of usual image-based studies. We target open-ended decoding, generalizing to new studies that might involve experimental conditions not yet probed in the available ones. We linked fMRI activation maps to a broad vocabulary characterizing more than 50 cognitive processes, by training machine learning models on heterogeneous statistical maps—from different experimental protocols and analysis pipelines—with unconstrained manual annotations. We then inferred mental processes from unseen studies, without any prior knowledge of the experimental setting or the relevant concepts.

This inference involves several challenges regarding neuroimaging-based knowledge accumulation, that we address in the current work. Specifically, we need to assess how fine descriptions of cognition can be captured from public repositories, and then decoded in a cross-study setting. For this, we had to address limitations of public data annotations: we proposed to achieve this by leveraging latent structure underlying those concepts. It was then possible to identify which concepts are harder to decode.

Such an endeavor has been made possible by the recent availability of aggregated cross-laboratory data. Specifically, all the image data exploited in this study come from NeuroVault^16^: the largest existing repository of fMRI statistical maps. As illustrated in Supplementary Fig. 1 , NeuroVault has undergone a significant growth in its first years and now hosts enough fMRI maps to allow for vast image-based meta-analyses.

### Contribution

Varoquaux et al.^7^ and Mensch et al^17^ have shown that image-based decoding across studies can build atlases mapping a diverse set of cognitive processes. Here we extend this prior work in three major ways. First, using all the data from NeuroVault, we trained models on ten times as many maps from more diverse sources. The training model hinges on an efficient data reduction mechanism, namely dictionary-based dimension reduction^18^, upon which powerful machine learning methods are easy to run and parameterize: we call this approach Neural Networks on Dictionaries (NNoD). Second, we used a larger vocabulary of concepts, obtained from Cognitive Atlas, that we extracted directly from the user-provided annotations available in NeuroVault. By doing so, we covered a broader spectrum of cognitive functions spanning more experimental conditions and subjects. This breadth enables a better assessment of the specificity of each brain region. Last, we assessed the usefulness of non-linear functional decoding models, using neural networks with few layers. For the first time in the field, thanks to the amount of data used in our experimental setting, those more expressive models yield better performance than linear models. Our conclusions are backed with strong quantitative evidence: training on more than 50,000 maps and 100 concepts, using a standard query system validation metric, we correctly rank among the first 10 best matches 66% of the relevant concepts of a map from an unseen study.

## Results

### Overview

In this work, we learn to *decode* fMRI statistical maps of the brain: given a brain map, we predict which mental conditions are associated with it. We did this by training supervised statistical models on data collected from the online platform NeuroVault^16^ (https://neurovault.org). Mappings from brain activations to mental concepts should not be specific to a particular study: instead they should hold across experimental paradigms. Therefore, we pooled data from many different neuroimaging studies in a cross-domain meta-analysis setting. Our evaluation method measures generalization to unseen studies.

**Figure 1:**
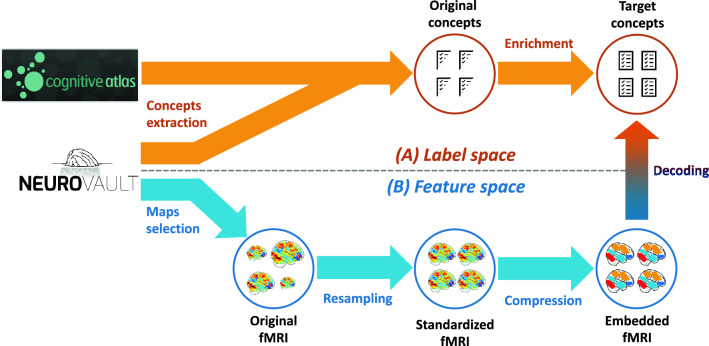
Approach for public data decoding. Part (**a**) extracts cognitive concepts from the annotations as labels and part (**b**) extracts the associated features from NeuroVault fMRI maps. (**a**) In the label space: we extracted any concept of Cognitive Atlas that we find in the annotations, then we enriched those using either imputation rules or similarities learned from an external source, which yielded several labels for each fMRI map. (**b**) In the feature space: we first selected the usable fMRI maps from NeuroVault, standardized them to a common resolution and brain mask, then we projected them on a dictionary of brain components extracted from a huge number of brain activation maps. Finally, we trained models to decode the concepts of (**a**) from the maps of (**b**).

In order to learn associations between brain activations and cognitive states, we had to: (i) collect fMRI maps and choose an appropriate vector-based representation for these data, (ii) associate cognitive labels with each brain map, given the available information, (iii) fit multi-label predictive learning models, and evaluate the performance of trained models. The full analysis pipeline is summarized in Fig. 1 and described in detail in Materials and methods section.

An important aspect of this study is that data are collected from an open online repository and are uncurated. In particular, the fMRI maps used in this work are not manually labeled: we must extract the labels, i.e. the supervision signal, from uncurated and weakly structured metadata. To do this, we matched concepts from the CognitiveAtlas ontology^1^ (https://www.cognitiveatlas.org) with the information in the metadata. This resulted in incomplete annotations for some images. Therefore, in a second experiment, we improved and enrich this initial labeling by leveraging the relations and hierarchical structure that bind cognitive concepts, and by applying a set of rules tailored to NeuroVault metadata. This enrichment was performed with simple rules that automate the process, and not on a case-by-case fashion.

### Best brain spatial representation

To represent the fMRI signal, we chose to rely on the DiFuMo set of probabilistic atlases, aka dictionaries https://parietal-inria.github.io/DiFuMo. This is based on the observation^17,19^ that, unlike intra-subject problems, inter-subject, and a fortiori inter-study, decoding problems almost always benefit from spatial compression, with only few exceptions^17^. We considered dictionaries of three different resolutions (128, 512 and 1024 components) to embed the original voxels activation in spaces of lower dimension. We also performed a stacking of those three embeddings to create multi-resolution representations that concatenate the above three. For those four representations, we can either keep all the dictionary loadings or only the positive values. We chose the dictionary resolution by training a baseline logistic linear model over the full training dataset. We used exact matches of Cognitive Atlas concepts in the metadata to label images, only removing those that are too rare or too correlated with another. We trained NNoD on 26,000 fMRI maps and a vocabulary of 96 concepts. We evaluated performances on the 6,500 maps from a test study, called Individual Brain Charting (IBC), using the same method of concepts extraction, where we find 37 of those concepts. We computed two metrics—one for classification, the Area under Curve (AUC) and one for *information retrieval*, the weighted recall at 10—for the different representations, based on the same train and test data.

**Figure 2:**
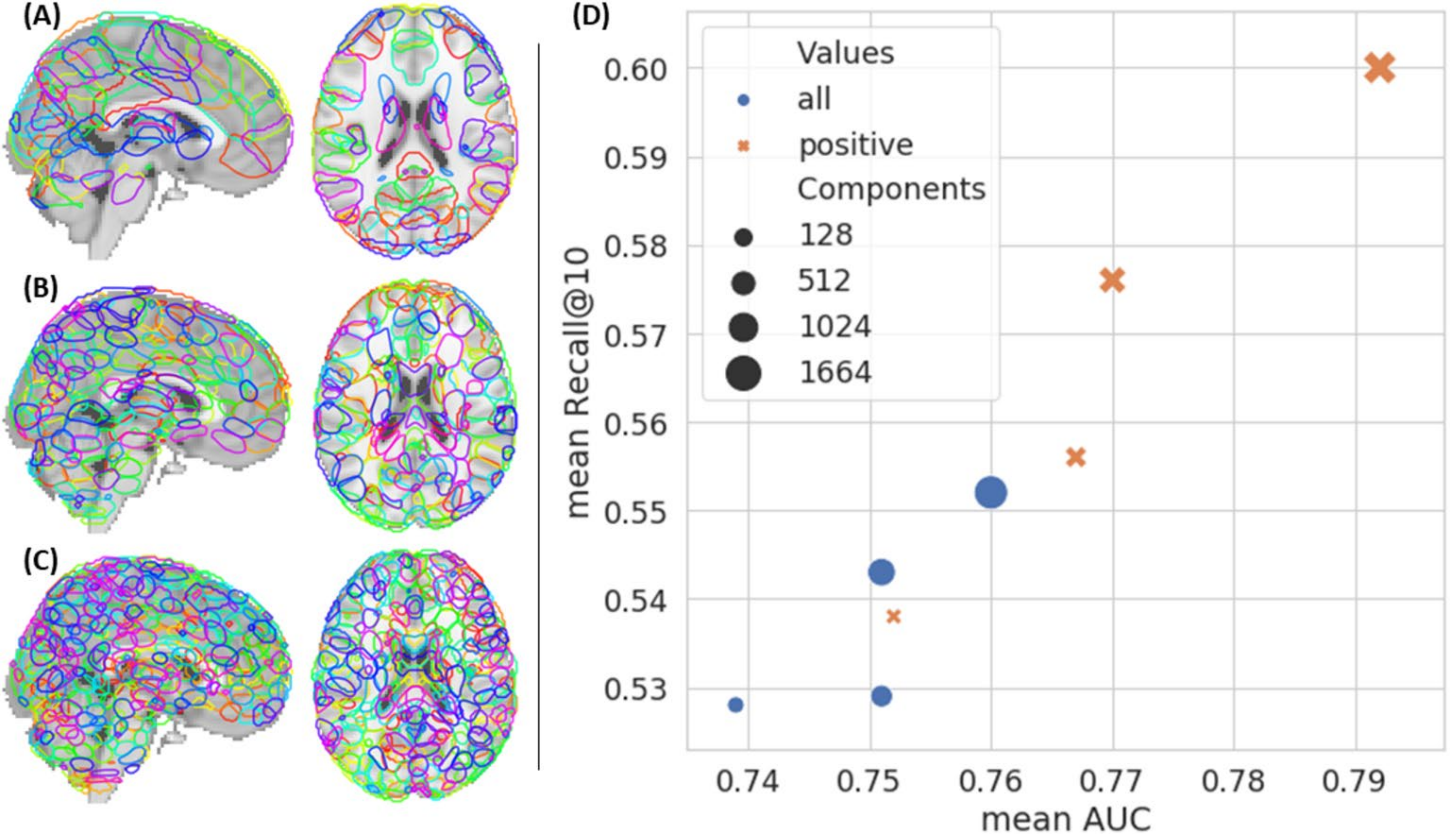
Preliminary identification of suitable image representations for large-scale decoding. (**a**), (**b**) and (**c**) respectively illustrate the components of the 128, 512 and 1024-components dictionaries. In each dictionary, the components are sparse but they have some overlap. In (**d**) we compare both the decoding performance for 37 concepts extracted from the original annotation, from projections on each dictionary as well as from the stacking of all projections, either for all values or just the positive ones.

Considering Fig. 2 we found that the best results for both metric are achieved by stacking the embeddings obtained by projecting maps on each dictionary: the compressed representation has $1024 + 512 + 128 = 1664$ dimensions. It is however worth mentioning that the performance gap between representations is limited, meaning that all of them capture at least some of the signal of interest. Moreover, we observed that taking only the positive part of the activation maps improves performance, the underlying intuition being that this reduces the impact of ill-specified control conditions.

### Decoding the concepts found in online annotations

**Figure 3:**
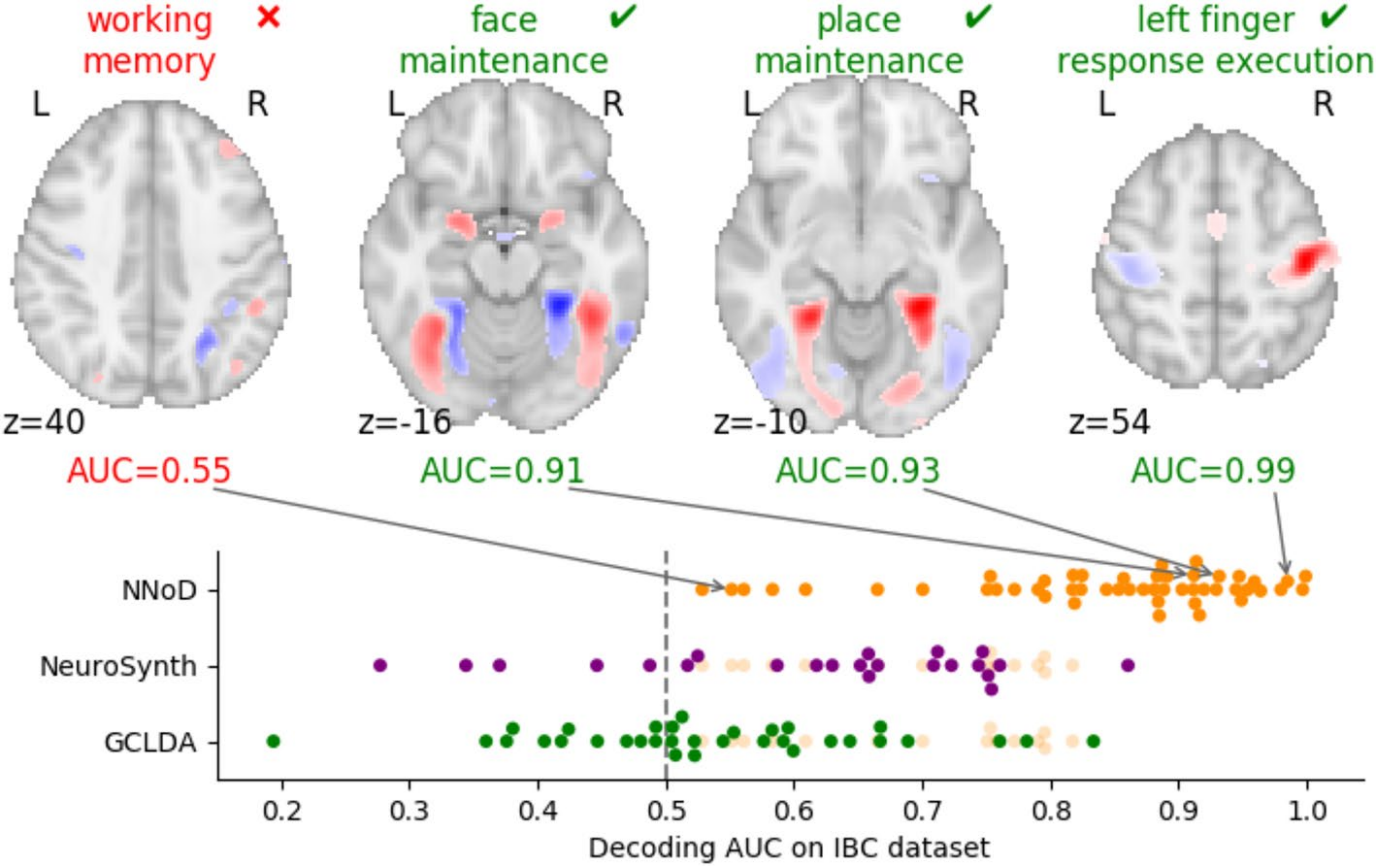
Decoding exactly-matched labels. We evaluated the AUC of the NNoD model on 37 labels matched in the IBC collection, after training it to decode 96 labels across collections. On the top, we show decoding maps for some example terms. Terms that are well decoded such as *place maintenance* have meaningful maps, whereas terms such as *working memory* whose neural correlates are poorly captured get low AUC scores. As the decoding maps do not have a meaningful scale, we threshold them arbitrarily at the 95th percentile for visualization. Using pre-trained GCLDA and NeuroSynth models, we compared NNoD results for the labels that also appear in the vocabulary recognized by these models (NNoD AUCs for terms in the vocabulary intersections are shown in light orange). Detailed scores for each label are represented in Fig. 4, showing that NNoD outperforms other methods for most labels.

Based on the setting described previously—26,000 fMRI maps and a vocabulary of 96 concepts for training, 6500 maps and 37 concepts from the IBC dataset (^20^, collection 4438), for testing—we show the Area under Curve (AUC) of NNoD and some decoding maps examples in Fig. 3. NNoD decoding achieved better-than-chance predictions for 35 out of 37 labels. The AUC is overall slightly higher than that of alternative meta-analytic decoding tools, such as GCLDA^11^ (using the implementation from https://github.com/tsalo/gclda, trained for 6755 concepts over the provided dataset of 11,362 studies) and NeuroSynth^10^ (implementation from https://github.com/neurosynth/neurosynth, trained for 3228 concepts over the provided dataset of 14,370 studies), but beware that the sets of concepts are different for these models. We provide a more detailed comparison in Table 1 below. The AUC metric seems also to capture the qualitative aspects of this decoding. Indeed terms for which the AUC is high, such as *left finger response execution*, tend to have plausible decoding maps, whereas terms that are not decoded above chance level, such as *working memory*, do not capture relevant brain regions in their decoding maps.

**Figure 4:**
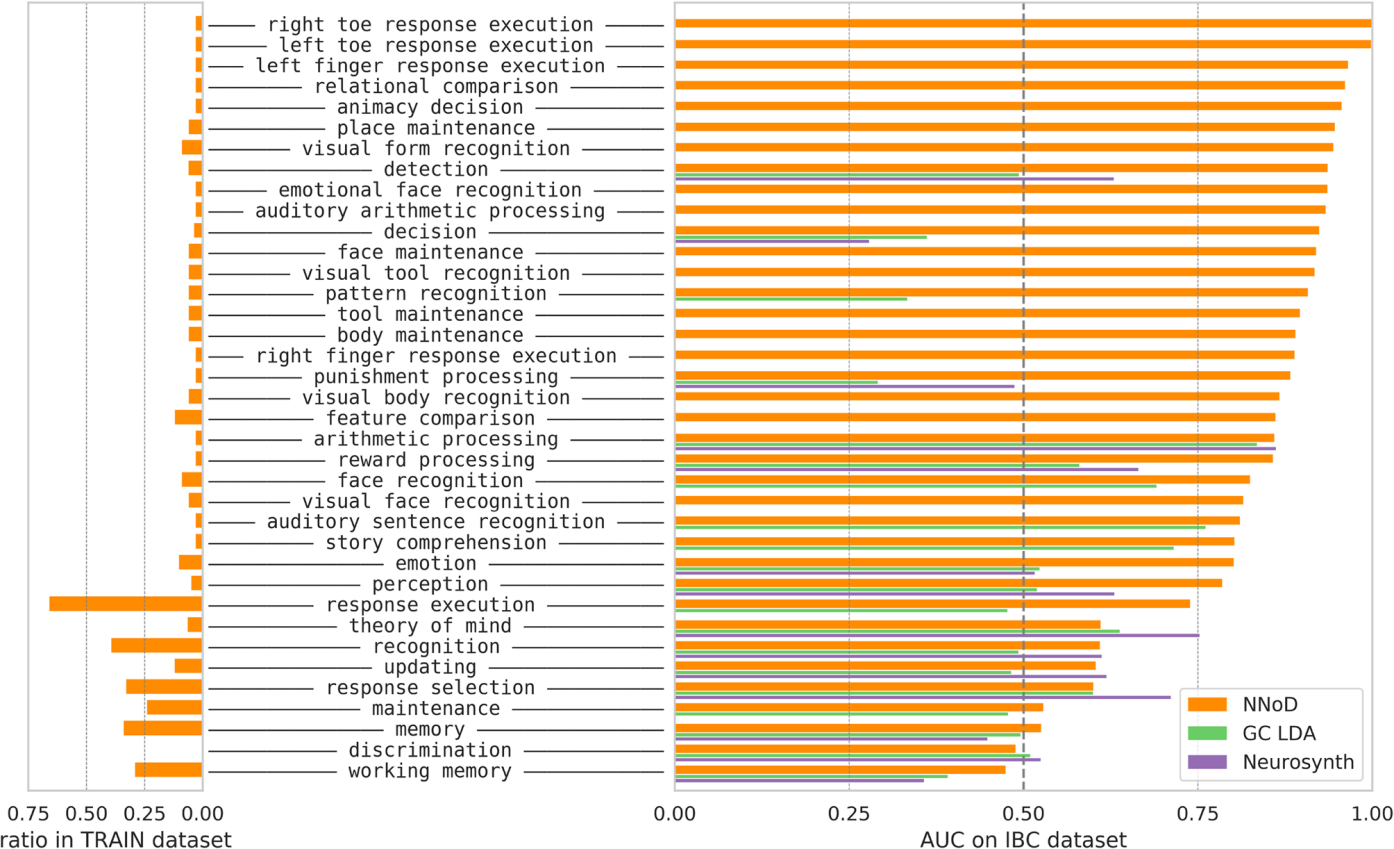
Performances on exactly-matched concepts. On the left, we display the label frequencies in the training set. On the right, we evaluated the AUC of NNoD on 37 concepts matched in the IBC dataset, after training it to decode 96 concepts from on all the other collections. Using pre-trained GCLDA and NeuroSynth models, we compared NNoD results for the concepts on which these models were also trained.

Figure 4 illustrates the detailed per-concept performance. Looking at the frequencies in the training dataset, we notice that the frequent ones are not better predicted. Somato-sensory concepts are particularly well decoded, as well as those related to mental arithmetics, and punishment/reward processing. We got worse results on high-level—and sometimes frequent—concepts like *working memory* and *theory of mind*. Other metrics and variants of the classification models are presented in Supplementary Table 4.

For comparison, Table 1 provides the performance of the GCLDA model from^11^ and NeuroSynth from^10^ on the same dataset for the concepts that are exactly matched in their vocabulary (listed in Supplementary Section A.8). It also displays the performance of the NNoD model on this subset of the predicted concepts.

**Table 1:** Models performance comparison on original concepts common with NeuroSynth or GCLDA.

Model	Common concepts	Model AUC	NNoD AUC
GCLDA	20	0.53	**0.73**
NeuroSynth	14	0.58	**0.71**

The main limitation of this first set of results is the paucity of concepts that are found across studies. In turn, this is due to the inconsistency of image annotations throughout NeuroVault.

### Decoding enriched concepts based on a cognitive ontology

To compensate for the limitations of publicly available annotations, we enriched and curated NeuroVault annotations for a second decoding experiment. We completed the dataset of labeled maps by adding rules to extract concepts from the largest collections, correct some common errors and abbreviations and extract some usual synonyms. We did not add any rule for the evaluation dataset to avoid biasing the reported metrics—the rules presented in this section are not applied to the test set.

Using these heuristics to enrich the label set, we extended the training dataset from 26,000 to 39,000, as well as the vocabulary from 96 concepts to 106 (despite merging 27 pairs of synonyms and removing highly correlated concepts). Among those concepts, 51 are matched or inferred from hypernymy rules in the test dataset annotations. Note that the number of test labels present in both training and test set increased because of the label inference rules. All the details regarding the label enrichment are presented in Supplementary Section A3.

**Figure 5:**
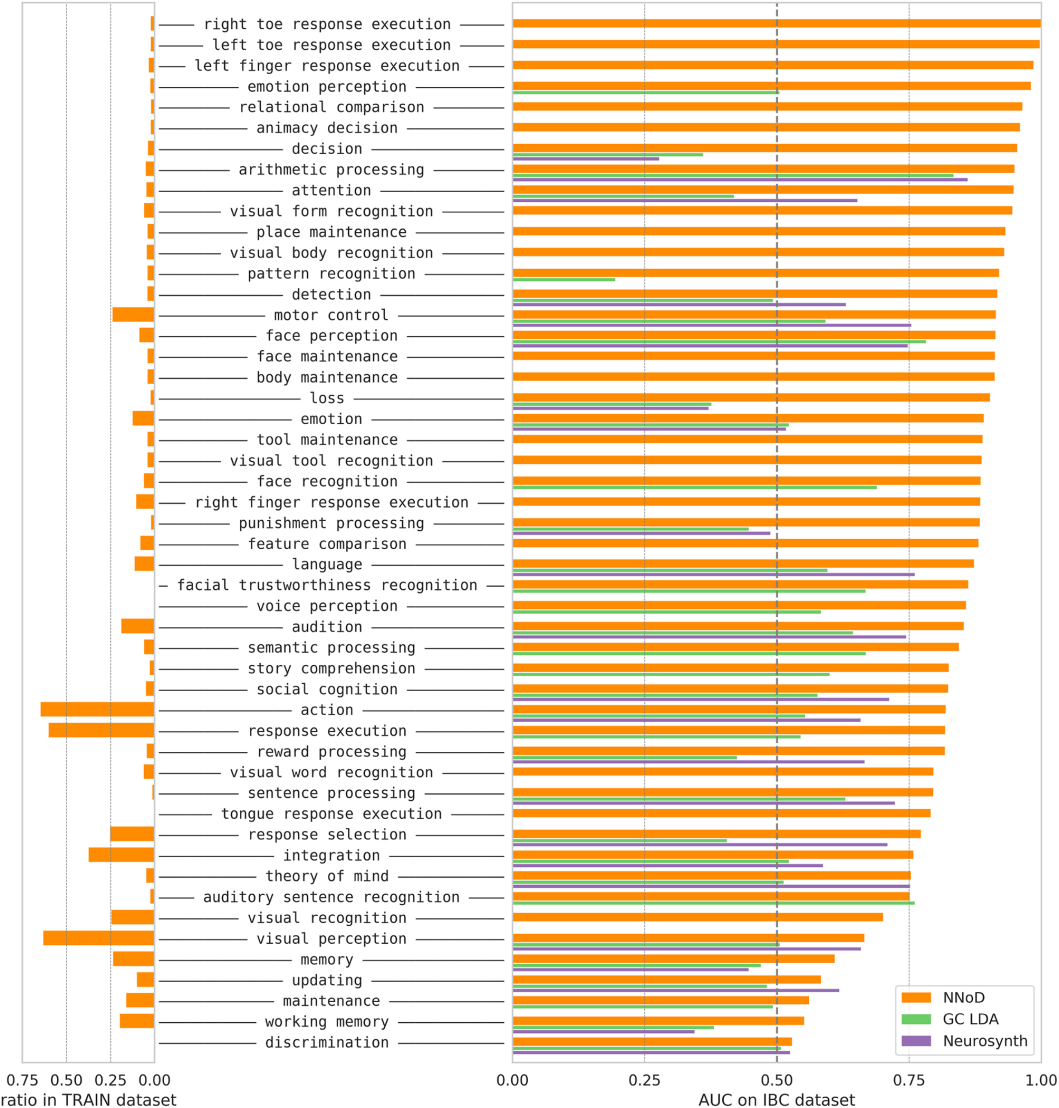
Decoding performance on enriched concepts. Compared with original labels decoding, after enriching the training dataset with heuristics, a similar model manages to decode more labels (51 in the test dataset) with an overall better accuracy.

Figure 5 provides the detailed per-concept results: we achieve better-than-chance predictions for 92% of the concepts considered. Other metrics and variants of the classification models are presented in Supplementary Table 5. We also performed experiments with different splits into train and test datasets, see Supplementary Section A.6 and Supplementary Figure 3.

Table 2 provides the comparison to NeuroSynth and GCLDA on the common concepts, showing again that NNoD architecture outperforms current alternatives.

**Table 2:** Models performance comparison on enriched concepts.

Model	Common concepts	Model AUC	NNoD AUC
GCLDA	31	0.54	**0.81**
NeuroSynth	23	0.62	**0.80**

### What brain structures support decoding?

A core question concerns what we indeed decode when we train models on such a dataset.

**Figure 6:**
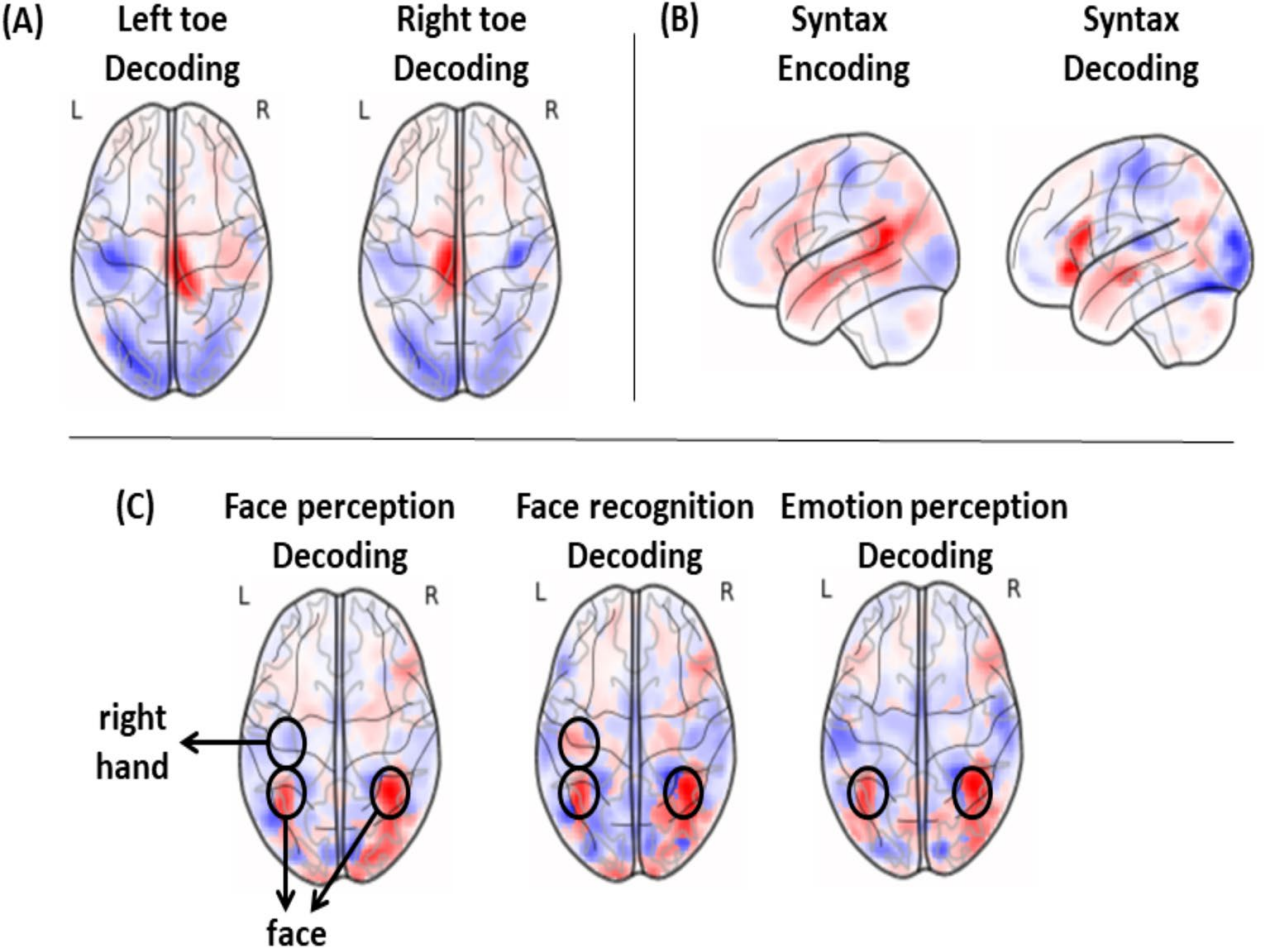
Illustration of some concepts encoding/decoding. Here we illustrate what the NNoD model learns from the data: (**a**) Left and right toe: For well known motor activities, NNoD properly identifies the corresponding area along the central sulcus^23^. It also uses the absence of activity in the other motor areas—here the absence of hand use—as motor functions are never jointly used in our experiments. (**b**) Syntax: Looking at the encoding of the syntactic activities, we observe that they generally involve Broca’s and Wernicke’s areas, as well as other brain areas related to stimuli and experimental actions. As in^7^, the decoding model discards common responses and focuses more on Broca’s area (Brodmann 44 and 45) which is consistent with the literature^21^. (**c**) Face perception, recognition and emotion: For *face perception*, NNoD correctly leverages activation in the FFA^22^. But it mostly identifies the concept of recognizing faces by the right-hand feedback commonly used by the subject. It also confuses *emotion perception* with *face perception*, missing e.g. the role of amygdala, as emotion perception is mostly tested by showing faces. See Supplementary Section A.7 for the similar illustrations of all the learned concepts.

For this we performed a sensitivity analysis on the decoding model—by automatic differentiation of the models outputs over their inputs—which made it possible to illustrate what are the main brain areas related to the concepts in the training dataset. In parallel, we considered the encoding maps of the training dataset—obtained by a linear regression of each brain component activation over the concepts, see supplementary section A.4 In Fig. 6 we give some representative examples. See Supplementary Section A.7 for encoding and decoding maps of all the learned concepts.

First, as seen in Fig. 6a, many concepts related to sensory or motor functions are uncontroversial. Second, in Fig. 6b we observe that the encoding of the syntactic tasks generally involves Broca’s and regions along the Superior Temporal Sulcus. By contrast, as in^7^, the decoding model discards common responses and focuses more on Broca’s area (Brodmann 44 and 45) which is consistent with the literature^21^. Finally, when considering *face perception*, in Fig. 6c NNoD correctly leverages activation in the FFA^22^, but it mostly identifies the concept of recognizing faces by the right-hand feedback commonly used by the subject. More worrying, it confuses *emotion perception* with *face perception*, missing the importance of amygdala activation, as emotion perception is mostly tested by showing faces in the datasets currently available on NeuroVault.

## Discussion

The present work establishes the feasibility of decoding brain maps at scale, as we obtained high prediction accuracy for brain activity decoding on the most comprehensive set of cognitive topics achieved so far. This decoding accuracy first demonstrates the value of the data stored in public repositories, in particular in NeuroVault. It also gives key insights for large-scale, cross-laboratory brain-image-based analyses of cognition.

In the experimental setting that we considered, NNoD vastly outperformed NeuroSynth and GCLDA and yielded a high AUC even from the original labels, despite the stated limitations. It has to be noted that both NeuroSynth and GCLDA were trained on far more concepts. Many of those are close to Cognitive Atlas concepts without exactly matching them. They might perform better with a different vocabulary.

On the considered feature and label spaces, non-linear models performed better than linear ones for decoding (best linear AUC = 0.82, best non-linear AUC = 0.84, see Supplementary section A.5 and Table 4). To our knowledge, even if this increase is minor, this is one of the first time those models prove useful to increase decoding performance in cognitive neuroscience.

Our approach also confirms that good decoding performance can be reached despite significant dimension reduction. This topic has already been studied in several previous works like^8,24^. We did not try training models on the original voxels maps as it would have required far more computing resource given the size of the dataset we used. On the other hand, it is noteworthy that stacking data at different resolutions improves prediction accuracy (see Fig. 2): it makes the model more robust to variations in the topographic layout of structures seen across experiments and datasets.

Starting from a vocabulary of more than 800 concepts, we end up training the NNoD model on 106 and evaluating them on only 51. While this is by far the most extensive image-based decoding study to date, this calls for some comments. Interestingly, successfully decoded terms not only involved sensory or somatotopic representations, but also high-level functions such as arithmetics, decision, relational comparison, different dimensions of language, social cognition plus emotion. This represents a clear improvement over prior work on open-ended decoding^7^, that only had 20 concepts, mostly tied to vision and motor representations.

First, we deal with a dataset whose annotations often use a wording different from the vocabulary we used. We experimented with some modern natural language processing techniques to automatically infer labels from the annotations. For example, we trained word embeddings on either big Internet corpora like Wikipedia or the whole PubMed literature, and tried automatically inferring labels using the similarities between the annotations and those concept representations. This did not improve results and we chose to revert to decoding the exactly-matched labels enriched with some heuristics. We also took care not to oppose synonymous concepts—either highly correlated in the available data or used indiscriminately from our observations—which caused us to group many labels. Finally, we were limited by the focus on some classical experiments in the available (and well annotated) collections. Despite the number of collections on NeuroVault, not that many different concepts are present in enough of them to allow for a proper out-of-sample decoding validation. Still, the enrichment we used illustrates how enforcing a consistent structure between labels allows for better decoding performance. In general, we can expect that annotations will improve with the use of more standard vocabulary^7^, or the reliance on relevant topics^25^. In the meanwhile, noise in the notations is at least partly overcome by aggregating more data.

A further goal of this work is to generalize to any dataset. In the present case, we have used IBC, as it allows validation on a large vocabulary, but we also show generalization to other datasets (see Supplementary Section A6). Such a decoding engine could be used quite systematically to assess the informative content of any candidate dataset.

Decoding performance varies between concepts, probably outlining some limitations of the public resources we used. We might indeed consider that some concepts from Cognitive Atlas cannot be decoded from functional brain images, either because of the available resolution or because they are not processes that can be inferred from neural activation patterns. As an illustration, Cognitive Atlas has a *wisdom* concept; one might wonder if it can really be identified from neural activations. Apart from this and the wording differences already observed, there is an issue of consistency in the way some concepts are used as annotations. Taking the example of the *working memory* concept in NeuroVault annotations, which we poorly decode, it is systematically used for tasks like contrasts between 2-back and 0-back tasks^26^, that are indeed the usual means of identifying this concept^27^. Yet, an inspection of these maps reveals that the expected fronto-parietal pattern was not found in all such images, making it hard to learn an actual representation for this. It is also used for other contrasts like 0-back versus baseline tasks, or contrasts involving tasks where the subjects have instructions to follow. Those inconsistencies threaten the quality of any automated meta-analysis. In the present case, the term is not identifiable. This issue illustrates the difficulty of crafting consistent labeling rules across the cognitive neuroscience community. Yet, such rules are clearly needed^28^.

This work trains models on maps from many studies—mixing z-maps, t-maps and beta-maps, i.e. raw regression coefficients that show the effect of experimental condition on the BOLD signal, but without statistical normalization—and evaluates performance on a study containing only z-maps. We tried training on only part of the dataset during some ablation tests. Removing the t-maps from the training dataset slightly decreased performance. Removing the univariate beta-maps had no impact (but the preparation pipeline excluded the maps with the most extreme values). Overall, we found that detailed and homogeneous annotations were more important than homogeneous and high resolution maps.

A difficulty in obtaining an accurate decoding model lies in the inconsistent baselines used in contrast-based fMRI: Each study uses a different baseline, that can be a control condition matched for all possible confounding aspects, or a low-level condition, such as fixating a cross. While these different baselines are justified by the purpose of each study that contributed the data, they make it harder to compare activation maps across experiments: while it is possible to account for the main effect of an experiment with an occurrence model, some images display more or less additional patterns that were not captured by the contrast baseline. This explains why taking only the positive part of the maps outperforms keeping all values, as it mitigates such baselines fluctuations, as can be seen in Fig. 2. An answer to that concern is that researchers deliver more contrasts per study, i.e. maps related to the main conditions, baselines and contrasts among them. Additionally, annotations associated with brain images should be more informative regarding contrast specification.

While we could confirm the usefulness of decoding to isolate the neural substrate of many cognitive concepts, already shown in^7^, we also uncovered some biases that deserve a word of caution. Indeed, some standard experimental protocols are frequently used to probe some specific cognitive processes. The model sometimes learns to recognize those protocols instead of brain areas more specific to the decoded mental processes. This improves model’s performance on standard experiments. For example, considering emotion perception, the NNoD model can leverage the relevant activation in the amygdala, but also the fact that emotion perception is usually probed by showing faces expressing different emotions. Unfortunately, this limits the generalization to new experimental protocols. Current NNoD models would perform poorly on a new experiment that would probe emotion perception by telling stories of people acting as happy or sad. The decoding of face perception, recognition and emotion shows that the method may not only infer the specificity of brain regions, but is also well attuned to decode some biases inherent to the field of cognitive neuroscience. In the case of complex concepts, such as theory of mind, it is also important to check that these are assessed in different ways (cartoon figure displaying interactions or not, stories involving beliefs and interpretation of other’s behavior or not etc.), both on the training and test set. Remaining biases are expected to be mitigated by the accumulation of data, which is however going to take time, given that consensus on how to map systems and analyze the results drifts slowly across time. A more intense result sharing commitment of the community would certainly help to solve this issue.

Altogether, we have demonstrated that modern machine learning methods trained on open data brain images can help decode broad vocabularies of cognitive concepts. They achieve good decoding performance from original uncurated annotations. They do so in a tractable way when proper dimension reduction techniques are used. The models used in this article can be trained in just a few minutes on a modern personal computer. Lightweight data curation, with simple rules on the annotations, improves decoding performance. We feel that such meta-analyses would yield more informative brain maps if the community could spend an additional effort toward systematic and consistent annotation rules following some ontology. The one we used to merge and infer some labels was built empirically from inconsistencies we noticed in Neurovault labeling. A more general ontology would be very helpful for meta-analyses but it requires some (difficult to achieve) consensus regarding relationships between concepts.

Although we pooled experiments from multiple laboratories, we also showed that decoding models can end up leveraging neural activation from specific experimental manipulations, obscuring the neural counterparts of cognitive processes of interest. Multiplying the ways of targeting mental processes, by constantly designing new protocols instead of replicating classical ones, appears as the best way to circumvent this issue.

A possible extension of this work is to combine it with automated coordinate-based meta-analyses based on the literature^10,12^: the latter would provide more comprehensive vocabularies, and guide the model in domains where cognitive labels could not reliably be inferred from annotations. Recent contributions^25^ have indeed shown that stable topics could be built from the literature, improving the robustness of image labelling.

## Materials and methods

### Data sources

NeuroVault^16^ is an online repository of fMRI statistical maps from cognitive studies (see Supplementary section A.2 ) designed to provide material for meta- and co-activation analysis. It contains annotated fMRI statistical maps grouped in collections, uploaded on a voluntary basis. Many of those collections are public. They can be explored and visualized via the website https://neurovault.org as well as downloaded via its API, with tools such as Nilearn^29^.

Cognitive Atlas^1^ is an online cognitive neuroscience knowledge base available at https://www.cognitiveatlas.org. It provides a description for more than 800 cognitive concepts and 700 experimental tasks, with some relationships between concepts as well as between concepts and tasks.

### fMRI statistical maps preparation and labeling

After a selection process excluding images of the wrong modality, those whose brain coverage was too low, that were too heavily thresholded or whose values are unreasonable for contrast-effects statistical images (t- or z-statistics), we kept 54,000 unique maps. All these maps represent individual effects. Details about this process are given in Supplementary section A.2.

We then extracted the cognitive concepts from Cognitive Atlas that we match exactly in the maps annotations, despite the wording differences between Cognitive Atlas and NeuroVault. Besides the particular case of HCP data (collection 4337), that were labeled according to Cognitive atlas, the extracted set of labels for 29 000 maps of NeuroVault is supplied online https://github.com/Parietal-INRIA/fmri_decoding/tree/master/extracted_labels.

As, the annotations of NeuroVault are mostly unconstrained and uncurated, some differ dramatically from Cognitive Atlas terms and there is no validity or homogeneity guarantee. This limits the number of studies that we could use in the analysis. Moreover, some concepts have hypernymy relationships: a task involving *auditory sentence comprehension* should involve at least *auditory sentence perception*, *auditory perception*, *perception*, *language comprehension* and *language* as well. Keeping such labels would lead to false negatives and more generally, inconsistencies in the labels. We thus crafted an enriched set of labels for 50 000 NeuroVault maps that solves this issue by simplifying and unifying the label set. It is supplied online https://github.com/Parietal-INRIA/fmri_decoding/tree/master/extracted_labels. More details about the labeling process and its issues are described in Supplementary section A.3.

### fMRI signal representation

fMRI statistical maps are high dimensional objects that aggregate many neurons’ activation in each voxel. Still, as illustrated in^30^, the activation localization for a given cognitive task can vary a lot across analysis pipelines.

To gain robustness against this variability, we project the signal on sets of brain regions that comprise highly correlated voxels, identified with a smooth parcellation method^31^. Such dictionaries of sparse spatial maps were trained using a stochastic online matrix factorization—SOMF—algorithm^32^ on 27 fMRI studies collected from OpenNeuro (https://openneuro.org). These dictionaries can be explored online: https://parietal-inria.github.io/DiFuMo. We transformed the matrix of resampled and masked voxels into a matrix $\mathbf {X}$ of loadings over those dictionaries by an orthogonal projection over components, using ordinary least squares regression (see Supplementary section A.2.4 ). Projecting the original maps on those dictionaries allows for a dimension reduction from $\approx 10^5$ voxels to $\approx 10^3$ components. This made the problem far more tractable, enabling more extensive explorations of models and parameters. Using these components is justified by the fact that they proved to be sufficient for a proper data reconstruction in^32^ and high decoding performance—especially compared to anatomical atlases—in^8^ and^24^.

We used dictionaries of three different resolutions (128, 512 and 1024 components) to embed the original voxels activation in spaces of lower dimension. We also performed a stacking of those three embeddings to create multi-resolution representations that concatenate the above three. For those four representations, we can either keep all the dictionary loadings or only the positive values. Taking the positive part of the loadings amounts to taking only the positive part of the maps, since the dictionary components are non-negative. This modeling choice is justified by the fact that we do not wish to take control conditions into account: negative activations are often related to the effect of the control condition. We chose the dictionary resolution by training a baseline logistic linear model over the full training dataset. We used exact matches of Cognitive Atlas concepts in the metadata to label images.

### Decoding models

#### System architecture

Decoding commonly relies on high-dimensional multivariate linear models^4^. Indeed, fMRIs are high-dimensional data and, aside from some rare large-scale studies such as HCP, they come with small sample sizes, i.e. they involve few subjects^33,34^. The whole analysis pipeline from the acquisition to the statistical map varies between studies^30^. Because of this low signal-to-noise ratio, non-linear models, that are more expressive, tend to overfit the noise in the data^8^.

**Figure 7:**
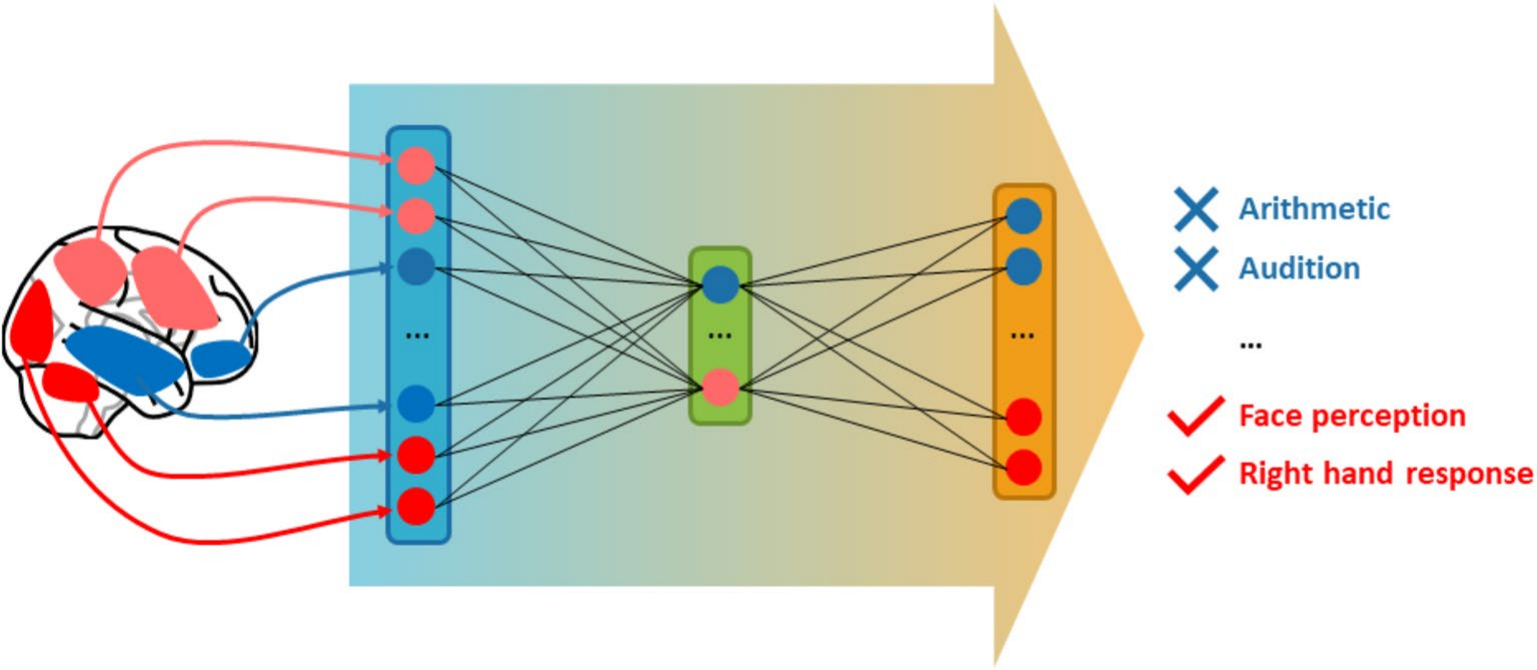
Explored ”NNoD” models. As inputs we used the brain components loadings for the studied contrasts (*in this example ”clicking when viewing a face” vs ”viewing something else”*). The hidden layers were used to increase the expressive power of the model by adding non-linearities and regularize it by controlling their width. We explored networks with 0 to 3 hidden layers. The output layer scores each considered label.

We explored shallow fully connected neural networks as decoding models. They are especially flexible in terms of regularization and allow to customize the loss function. We trained them to jointly score all the concepts of the vocabulary extracted from annotations based on the compressed representation (dictionary loadings) of each map, as shown in Fig. 7. These models are related to the factored logistic regressions of^35^, but differ in the use of an alternate basis for the initial projection and deeper models for classification. In the following, we call these models ”NNoD” for ”Neural Network over Dictionary”. We used up to 3 hidden layers. For the hidden units, we considered the identity activation function (resulting in a linear model) and the rectifier activation function $z \mapsto \max (z, 0)$. Output units, regularization, and model selection are discussed in the rest of this section. The technical implementation is available on https://github.com/Parietal-INRIA/fmri_decoding. It includes the full pipeline to fetch, prepare and decode the data. It was developed in Python 3.6 and mostly relies on Nilearn 0.5^29^, Scikit-learn 0.21^36^ and PyTorch 1.0^37^.

#### Loss function

The form of the loss function depends on how we model the occurrence of labels. A first possibility is to treat each label as a separate binary classification task. A second option is to treat the problem as a multi-output classification task, and learn to predict a categorical distribution over all possible labels. In both cases, the corresponding model is trained following the empirical risk minimization principle. We denote $f_{\theta }$ the function learned by the neural network up to the output activation function, which is parameterized by $\theta$, the learned coefficients of the model. For example, in the simplest case where there are no hidden layers – i.e. logistic regression, $f_{\theta }$ can be written:

1 $$\begin{aligned} f_{\theta }(\varvec{x}) = \varvec{W} \, \varvec{x} + \varvec{b} \; , \end{aligned}$$

where $\varvec{W} \in \mathbb {R}^{|L| \times p}$ and $\varvec{b} \in \mathbb {R}^{|L|}$ are the coefficients of the model and $\theta = \{\varvec{W}, \varvec{b}\}$. Then, the model’s prediction is:

2 $$\begin{aligned} \hat{\varvec{y}} = g(f_{\theta }(\varvec{x})) \, , \end{aligned}$$

where *g* is the output activation function and $\varvec{x} \in \mathbb {R}^p$ is the input vector of dictionary loadings.

#### Binary classification

We can consider labels separately, and treat the computation of a score for each label as a different classification task. In this case the output distribution for each label is a Bernoulli distribution and we use sigmoid ($\sigma$) output units. We denote the loss $\mathcal {L}_{bin}$ and apply a regularization over the network weights $\mathcal {R}(\theta )$ as in Eqs. (3), (4) and  (5).

3 $$\begin{aligned} \widehat{\mathbf {y}} = f_{bin}(\mathbf {x})= &  \sigma (f_{\theta }(\varvec{x})) \end{aligned}$$

4 $$\begin{aligned} \sigma&:&z \mapsto \frac{1}{1 + \mathrm {e}^{-z}} \end{aligned}$$

5 $$\begin{aligned} \mathcal {L}_{bin}(\mathbf {y}, \widehat{\mathbf {y}})= &  - \sum _{l \in L} \left( \mathbf {y}^l \log (\widehat{\mathbf {y}^l}) + (1 - \mathbf {y}^l) \log (1 - \widehat{\mathbf {y}^l}) \right) + \mathcal {R}(\theta ) \; . \end{aligned}$$

In Eq 3, $\sigma$ is applied element-wise.

#### Multi-output classification

A slightly different approach is to introduce a tighter coupling between the labels and consider that they are actually *competing*. In this case, instead of producing one Bernoulli parameter for each label, the model produces the parameter of a categorical distribution over all the labels: a vector in the probability simplex of $\mathbb {R}^{|\mathcal {L}|}$, where $|\mathcal {L}|$ is the number of possible labels. In this case, a softmax function ($\tau$), defined as in Eq. (6) is applied to the output layer of the network.

6 $$\begin{aligned} \tau (\varvec{z})^l = \frac{\exp (\varvec{z}^l)}{\sum _l \exp (\varvec{z}^l)} \, . \end{aligned}$$

The loss of these multinomial logistic models, $\mathcal {L}_{mult}$, is defined as in Eqs. (7) and  (8).

7 $$\begin{aligned} \widehat{\mathbf {y}} = f_{mult}(\mathbf {x})= &  \tau (f_{\theta }(\varvec{x})) \end{aligned}$$

8 $$\begin{aligned} \mathcal {L}_{mult}(\mathbf {y}, \widehat{\mathbf {y}})= &  - \sum _{l \in L} \left( \frac{\mathbf {y}^l}{\sum _{k \in L}\mathbf {y}^k} \log (\widehat{\mathbf {y}^l}) \right) + \mathcal {R}(\theta ) \end{aligned}$$

#### Regularization

We used an elastic-net penalty^38^ on the model coefficients as regularization $\mathcal {R}$. This regularization penalizes a combination of the squared norm and sum of absolute values of the parameter vector. Moreover, we applied dropout^39^ on the input and hidden layers. We applied the same penalty and level of dropout on all layers.

#### Model selection

We selected the amount of regularization (2 parameters for the elastic-net penalty), the amount of dropout (45% or 0%), type of hidden unit and width of the hidden layers (250 or 300 neurons) with an inner loop of cross-validation. To do so, we performed a grid search for these hyper-parameters on the training data. For the validation, we used a single-fold scheme, using the IBC dataset on the initial setting, i.e. without label enrichment. Given that this setting worked similarly or better for different data (other test sets and label enrichment), we conclude that there was no overfitting on hyper-parameter selection.

The following parameters were thus selected: The classifier is a two-layer perceptron using the stacked dictionary loadings. Following the results presented in Fig. 2, the inputs are the stacked loadings for all 3 dictionaries and have dimension $1024 + 512 + 128 = 1664$, the hidden layer has width 300, and a dropout of 0.2 is applied on the hidden layer (i.e.  elements have a 0.2 probability of being set to 0). The same penalty was eventually applied on $\ell _1$ and $\ell _2$ norms of coefficients and is set to 0.001. The activation function for hidden units was the rectifier. Classification scores presented in the results section use a held-out test set.

### Decoding performance evaluation

We strove to jointly decode as many concepts from Cognitive Atlas as possible and focus on generalization across experimental protocols. As in^7^, this objective is framed as a multi-label classification problem, since several concepts are relevant for most maps. However, compared to this previous work, we intend to decode a broader vocabulary comprising all the concepts we could automatically extract from the annotations.

We have a varying number of ground-truth labels between maps, depending on the annotations and the labels extraction method. We therefore only used metrics that are agnostic to this total label cardinality and to their distribution over samples. No decoding study has used the same set of concepts. Still, some works like^7,10,17^ and^11^ have targeted partially overlapping sets. We chose the metrics to ensure that they gauge the performance of models for all considered concepts and do not boil down to capturing a good prediction of the most frequent terms only. This is important because some common concepts—such as auditory or visual perception—do not pose a decoding challenge. Also, we wanted to achieve a proper decoding of left-out studies, which might correspond to a new protocol inducing label shifts compared to the training data.

Consequently, to assess the decoding performance, we used two different metrics that are both independent from labels prevalence and are computed on a per-label (instead of per-sample) basis.

#### Weighted recall at k (WR@k)

For each label, we estimated the probability that, given a map for which this label is true, the decoder would rank it in the top-k labels. Then, we averaged this score across labels to obtain a single score.

The usual *recall at k* is averaged over the samples and not the labels. We call this metric *weighted recall at k, WR@k* as the recall over rare labels is given more importance than the recall for common (and sometimes trivial to decode) labels. It is presented in Eq (9):

9 $$\begin{aligned} {{\,\mathrm{WR@k}\,}}= \frac{1}{|L|} ~ \sum _{l} \frac{\left| \{ {{\,\mathrm{top_k}\,}}(\widehat{y}^l_i), \; y_i^l = 1 \}\right| }{\{i, y_i^l = 1\}} \; , \end{aligned}$$

where |*L*| is the total number of labels and ${{\,\mathrm{top_k}\,}}(\hat{y}_i^l)$ indicates that the prediction $\hat{\varvec{y}}_i$ ranks label *l* among the first *k*. This metric is interesting as it is easy to interpret and is typically relevant for a search engine that would decode candidate brain maps. Still, it does not fully reflect the false positives rate, the chance level depends on the set of labels and this metric is sensitive to the choice of parameter *k*. We therefore considered an additional metric.

#### ROC AUC

While being more difficult to interpret, the area under the receiver operating characteristic—ROC AUC, designated by AUC in the following—summarizes the rates of true and false positives better. It is also better suited to comparing approaches on a per-label basis, as the score for a given label does not depend on other labels. For a given model and label, if we draw a random map for which this concept is true and another for which it is not, the AUC can be understood as the probability of the model scoring the label higher for the first map than for the second. Once again, we averaged this metric over labels with uniform weights as presented in Eq (10):

10 $$\begin{aligned} {{\,\mathrm{AUC}\,}}= \frac{1}{\left| L\right| } \sum _{l \in L} \mathbb {P}(\widehat{y}^l_i > \widehat{y}^l_j \; | \; y_i^l = 1,\; y_j^l = 0) \; . \end{aligned}$$

In the following, we rely on the AUC for model selection.

#### External validation

When comparing methods, we used the maps from the IBC study for validation. Note that this NeuroVault collection has been contributed by some co-authors of the manuscript, but was set up independently from the need of the present experiment. It presents several advantages for our benchmarks. Indeed, it currently provides per-subject maps that cover 12 broad experimental protocols including, but not limited to, localizing tasks replicated from HCP. These tasks involve tens of different concepts from various cognitive domains and are annotated using concepts from Cognitive Atlas. IBC is thus an extensive and reliable validation dataset.

Moreover, we performed other validation experiments on different datasets. The corresponding results are displayed in Supplementary Figure 3 and are comparable with those obtained using collection 4438 as a validation set. It should be noted that the set of concepts that can be tested depends on the left-out collections, which makes it hard to compare accuracy across different folds.

#### Alternatives methods

GCLDA^11^ is a generalization of the correspondence-LDA model^40^, which is an unsupervised learning model used for modeling multiple data-types, where one data-type describes the other. Concretely, GCLDA identifies topics associated with a spatial probability distribution that captures the extent of function brain region, based on a Gaussian mixture model, and with a probability distribution of linguistic features that capture the cognitive function of regions. We used it to infer cognitive features from topographies, using the publicly available implementation .

The NeuroSynth meta-analysis platform^10^ also contains a built-in decoding model based on a Naive Bayes inference scheme, that we also considered as alternative procedure.

## Supplementary information

Supplementary Information 1.
