# Supplementary material for: Comprehensive decoding mental processes from Web repositories of functional brain images
Source: Sci Rep. 2022 Apr 29;12:7050. doi: 10.1038/s41598-022-10710-1 (PMC9054752; doi:10.1038/s41598-022-10710-1)
Supplement: Supplementary file 1 — Supplementary Information 1. [file 41598_2022_10710_MOESM1_ESM.pdf]

A Supporting information

A.1 Growth of NeuroVault

Supplementary Figure 1 illustrates the evolution of the total number of images hosted on NeuroVault from its creation to the end of 2020.

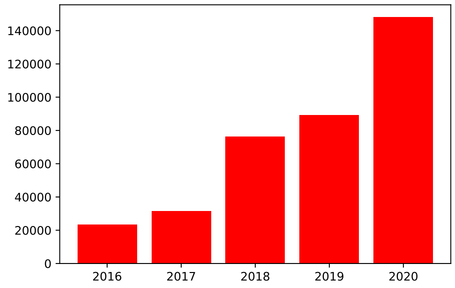

Supplementary Figure 1. NeuroVault base growth. This figure illustrates the evolution of the total number of images hosted on NeuroVault from its creation to the end of 2020.

A.2 fMRI statistical maps selection and preparation

A.2.1 fMRI maps from cognitive experiments.

From a meta-analytic perspective, functional MRI cognitive experiments are designed to study cognitive concepts ranging from stimuli perception and motor functions to high-order functions like working memory. They do this by recording the brain activity of subjects in controlled conditions: subjects are exposed to stimuli or asked to performs tasks that involve the studied concept, like identifying pictures containing faces, and sometimes related tasks whose only difference is the absence of the concept of interest, like identifying any other picture. Standard tasks are used by different laboratories to study some set of concepts.

After standard preprocessing, including motion correction and alignment to a standard brain template, the blood-oxygen-level-dependent (BOLD[41]) signal acquired during an experiment in each voxel reflects the hemodynamic response to the conditions. These time courses are then subject to regression analysis, with a model including the studied concept and various confounds, either for single subjects (first level analysis) or for a cohort of subjects (second level analysis). To produce the desired contrast, a statistical test — usually a t-test — is then performed between the coefficients of the studied concept and those of either a related concept or a baseline.

For a given experiment, those step yields 3D maps of voxels with a resolution of $\approx 10mm^3$, either per-subject or for the whole experimental cohort ($\approx 10^5$ useful voxels in the corresponding gray-matter mask).

A.2.2 NeuroVault maps

NeuroVault mainly aims at storing unthresholded maps of whole human brains. Still, many statistical maps are thresholded, some maps only provide activation for a limited region of interest (for example a single hemisphere), some are not statistical maps (for example brain atlases or masks), or not from human adult brains (for example young baboons). Some of the statistical maps are not contrasts between conditions or do not have a Gaussian or Student distribution as null distribution, and thus display different value distributions than other contrast maps. These outliers are either identified from the metadata or from the maps' values. We illustrate some of them in section A.2.3.

As of January 2020 and after removing exact duplicates, we retrieved 62,915 maps with the *fMRI-BOLD* modality among the 74,132 brain images hosted on NeuroVault, grouped as 1,136 collections from many — sometimes anonymous — sources and adding up to more than 60 gigabytes of compressed data. The number of maps in a collection varies from 1 to 18,070 —the

latter being the Human Connectome Project[42] (HCP), the largest fMRI cognitive study to date. We started by excluding images of the wrong modality, those whose brain coverage was too low, that are too heavily thresholded or whose values are unreasonable for contrast-effects statistical images (t- or z-statistics). This step yielded 54,000 unique maps.

Some of the maps we kept were missing part of the brain (at most 35%), often in areas that were not of interest for the considered experiment. We tried to impute those missing areas at component level, either by the median value or by sampling among the other maps, but this did not improve the decoding performance with respect to setting them to zero.

A.2.3 NeuroVault anomalies

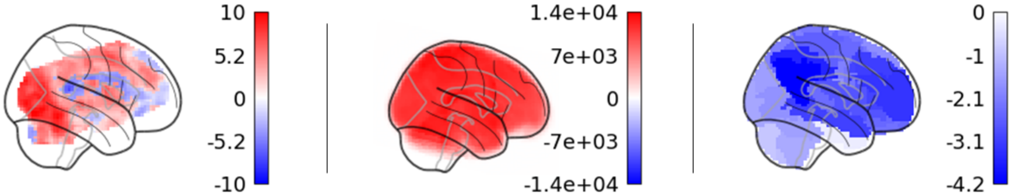

Illustration of some map anomalies. Those 3 maps are labeled as regular fMRI statistical maps in NeuroVault but the first lacks 40% of the brain volume, the second has unreasonably large values and the third has only negative values.

A.2.4 Projection of fMRI data on dictionaries

Orthogonal projection over a dictionary. To compute the code $\mathbf{X} \in \mathbb{R}^{n*c}$ over the components of dictionary $\mathbf{D} \in \mathbb{R}^{c*v}$ from the original voxel maps $\widetilde{\mathbf{X}} \in \mathbb{R}^{n*v}$ efficiently, with n the number of maps and v the number voxels in the grey matter mask, we multiplied the Moore-Penrose pseudo-inverse matrix of the dictionary (noted $\mathbf{D}^{\dagger}$) with the maps matrix, which simply yields Eq (12) as the dictionary's components are linearly independent (but not orthogonal).

$$\mathbf{X} = \widetilde{\mathbf{X}}\mathbf{D}^{\dagger} \quad (11)$$

$$= \widetilde{\mathbf{X}}\mathbf{D}^{T}(\mathbf{D}\mathbf{D}^{T})^{-1} \quad (12)$$

These vectors of loadings over the brain components $\mathbf{X}$ can then be simply re-projected over the voxel space as $\widehat{\mathbf{X}}$ in Eq (13). For example, this is useful to visualize the brain areas that are more significant for a statistical model trained on the component space as in Supplementary Section A.7.

$$\widehat{\mathbf{X}} = \mathbf{X}\mathbf{D} \quad (13)$$

A.3 Labeling strategies

A.3.1 Basic labeling

We started by extracting the cognitive concepts from Cognitive Atlas that we matched exactly in the maps annotations, despite the wording differences between Cognitive Atlas and NeuroVault. For many images, the control conditions are not provided or cannot be reliably extracted from the metadata. Therefore for consistency we removed the control conditions for all the images. We removed from the annotations the text that is obviously related to a control condition (appearing after a "versus", "vs", ">"... in the *contrast_definition* or *name* fields). We also improved the annotations of the Human Connectome Project (HCP) study[42] (collection 4337) with previously existing rules that are reproduced in the repository (https://github.com/Parietal-INRIA/fmri_decoding). We removed the labels that are very rare ($<$ 10 occurrences) as well as those whose occurrence is too correlated ($|corr| > 0.95$), as learning on scarce or overly correlated data may not yield meaningful results. The extracted set of labels for 29 000 maps of NeuroVault is supplied online https://github.com/Parietal-INRIA/fmri_decoding/tree/master/extracted_labels.

A.3.2 Enriched concepts

An exploration of Neurovault annotations illustrates some of the challenges in assigning cognitive labels by matching of Cognitive Atlas concepts found in the metadata.

First, Cognitive Atlas names concepts using a specific vocabulary, with denominations that are often quite long. On the contrary, the annotations of NeuroVault are mostly unconstrained and uncurated. Some of them do not contain enough information to make sense of the experimental protocol and the studied cognitive concepts. For the others, the wording can differ a lot from Cognitive Atlas' and there is no validity or homogeneity guarantee. For example, many studies use a specific wording that differs from Cognitive Atlas, using "right hand", "r.hand" or even "RH" instead of *right hand response execution*. This limits the number of studies that we can use in the analysis.

Second, the annotations in the maps include some spurious labels, as the annotations in some major collections use words in their annotations that are related to the concept of interest of the overall experiment, instead of the exact contrast of the map. For example, 786 maps corresponding to *shape recognition* appear with the concept name *emotion* in their annotations, as the corresponding study uses them as a baseline in an emotional task. This introduces false-positives in the labels.

Last, there is structure between these concepts that we do not leverage in the first experiment. Some concepts have hypernymy relationships: a task involving *auditory sentence comprehension* should involve at least *auditory sentence perception*, *auditory perception*, *perception*, *language comprehension* and *language* as well. Some are also very close, even synonymous or at least often used interchangeably in NeuroVault annotations. Automatically discriminating them from open data seems unreasonable: we do not expect that the use of *audition* in NeuroVault annotations conveys a different meaning that the use of *auditory perception*. This last issue causes false negatives in the target labels.

The enriched set of labels for 50 000 NeuroVault maps is supplied online `https://github.com/Parietal-INRIA/fmri_decoding/tree/master/extracted_labels`.

Considering the relationships between concepts, we also define an ontology[43] for the encountered concepts – rather than using the relationships from Cognitive Atlas that we found too incomplete (see section A.3.3). This heuristic directed graph includes 27 synonymy and 158 hypernymy rules. A small part of this graph is illustrated in Supplementary Figure 2. We also applied those rules on the evaluation dataset to get a consistent structure between the labels for all the data. The list of synonymy and hypernymy rules are presented in tables 1 and 2.

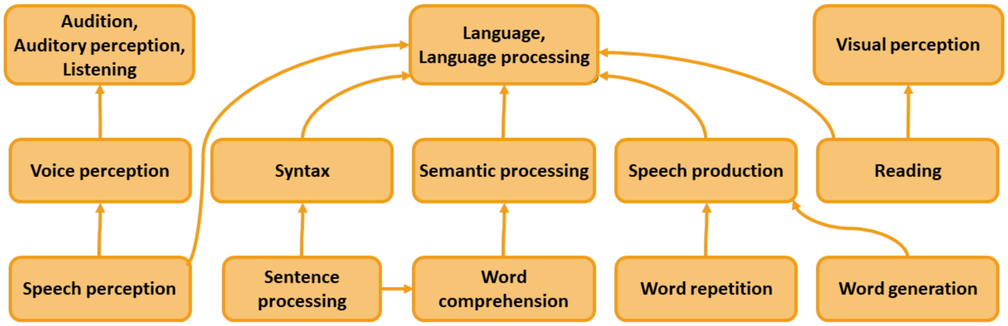

Supplementary Figure 2. Missing concepts inference graph. Here we illustrate a part of the cognitive ontology used in this work, for some concepts related to language. The arrows show the inference directions. For example, whenever we assign the concept *reading*, we also add *language* and *visual perception*.

Supplementary Table 2. Hypernymy rules. Additional labels associated to initial vocabulary.

label	associated labels

response bias	response selection
response conflict	response selection
response execution	action
response selection	action
auditory coding	audition
auditory tone detection	audition
auditory tone discrimination	audition
voice perception	audition
audition	perception
mathematical reasoning	arithmetic processing
mental arithmetic	arithmetic processing
numerical comparison	arithmetic processing
attention shifting	attention
fixation	attention
interference control	attention
spatial attention	attention,spatial ability
search	attention
visual attention	attention,visual perception
response inhibition	inhibition
behavioral inhibition	inhibition
attention	cognitive control
inhibition	cognitive control
proactive control	cognitive control
selective control	cognitive control
task switching	cognitive control
decision certainty	decision making
decision under uncertainty	decision making
risk aversion	risk
risk processing	risk
risk seeking	risk
anticipation	planning
uncertainty	planning
decision making	decision
risk	decision
planning	decision
emotion recognition	emotion perception,recognition,visual perception
emotional suppression	emotion regulation
emotional enhancement	emotion regulation
fear	negative emotion
stress	negative emotion
arousal	emotion
emotion perception	emotion
emotion regulation	emotion,cognitive control
emotional intelligence	emotion,social cognition
negative emotion	emotion
categorical perception	categorization
pattern recognition	recognition,visual perception
discrimination	recognition
association	integration
categorization	integration
error detection	integration
feature comparison	integration

phonological comparison	integration
recognition	integration
relational comparison	integration
auditory sentence recognition	semantic processing,audition
story comprehension	sentence processing
reading	semantic processing,visual word recognition
semantic categorization	semantic processing,categorization
semantic priming	semantic processing,priming
sentence processing	word comprehension, syntax
word comprehension	semantic processing
word maintenance	semantic processing,working memory
word recognition	semantic processing
overt naming	naming
naming	speech production
word generation	speech production
word repetition	speech production
syntax	language
semantic processing	language
speech production	language
speech perception	language,voice perception
instrumental learning	memory acquisition
reinforcement learning	memory acquisition
rule learning	memory acquisition
concept learning	learning
autobiographical memory	memory
episodic memory	memory
forgetting	memory
learning	memory
memory acquisition	memory
memory retrieval	memory
memory trace	memory
spatial memory	memory
motor sequence learning	motor planning
left finger response execution	motor control,response execution
left hand response execution	motor control,response execution
left toe response execution	motor control,response execution
right finger response execution	motor control,response execution
right hand response execution	motor control,response execution
right toe response execution	motor control,response execution
tongue response execution	motor control,response execution
motor planning	motor control,planning
movement	motor control
effort	pain
pain habituation	pain
conceptual priming	priming
detection	attention,recognition
causal inference	inference
analogical reasoning	reasoning
inference	reasoning
introspection	reasoning
judgment	reasoning
logic	reasoning

meaning	reasoning
mental representation	reasoning
monitoring	reasoning
problem solving	reasoning
animacy decision	animacy perception
animacy perception	social cognition
social inference	social cognition,inference
social norm processing	social cognition
theory of mind	social cognition
localization	spatial ability
mental rotation	spatial ability
spatial localization	spatial ability
spatial ability	visual perception
potential monetary reward	reward processing, anticipation
reward anticipation	reward processing, anticipation
reward valuation	reward processing
loss anticipation	loss, anticipation
loss aversion	loss, anticipation
potential monetary loss	loss, anticipation
punishment processing	loss
reward processing	valence
loss	valence
facial trustworthiness recognition	face perception
face recognition	face perception,recognition
visual body recognition	visual recognition
visual form recognition	visual recognition
visual object detection	visual recognition,attention
visual object recognition	visual recognition
visual pattern recognition	visual recognition
visual place recognition	visual recognition
visual tool recognition	visual recognition
visual word recognition	visual recognition
face perception	visual perception
mental imagery	visual perception
motion detection	visual perception,attention
object perception	visual perception
visual localization	visual perception,spatial ability
visual memory	visual perception,memory
visual orientation	visual perception,spatial ability
visual recognition	visual perception,recognition
visual search	visual perception,attention
visual working memory	visual perception,working memory
body maintenance	maintenance
face maintenance	maintenance
place maintenance	maintenance
object maintenance	maintenance
string maintenance	maintenance
tool maintenance	maintenance
visual object maintenance	maintenance
maintenance	working memory
procedural memory	working memory
set shifting	working memory

spatial working memory	working memory,spatial ability
updating	working memory
working memory retrieval	working memory

Supplementary Table 3. Matching rules. Labels matching done by regular expressions.

<table>
<tr><th>collection</th><th>regular expression</th><th>label</th></tr>
<tr><td>.*</td><td>left.*hand|l[\s_]hand</td><td>left hand response execution</td></tr>
<tr><td>.*</td><td>right.*hand|r[\s_]hand</td><td>right hand response execution</td></tr>
<tr><td>.*</td><td>left.*finger|l[\s_]finger|left.*click</td><td>left finger response execution</td></tr>
<tr><td>.*</td><td>right.*finger|r[\s_]finger|right.*click</td><td>right finger response execution</td></tr>
<tr><td>.*</td><td>left.*toe|l[\s_]toe|left.*foot|l[\s_]foot</td><td>left toe response execution</td></tr>
<tr><td>.*</td><td>right.*toe|r[\s_]toe|right.*foot|r[\s_]foot</td><td>right toe response execution</td></tr>
<tr><td>.*</td><td>[\s_]face|^face</td><td>face perception</td></tr>
<tr><td>.*</td><td>audit|listen|hear|sound|music|noise|voice|acoust</td><td>audition</td></tr>
<tr><td>.*</td><td>visual|view|watch|picture|movie|moving</td><td>visual perception</td></tr>
<tr><td>.*</td><td>rule|wm</td><td>working memory</td></tr>
<tr><td>.*</td><td>visual word</td><td>visual word recognition</td></tr>
<tr><td>.*</td><td>read</td><td>reading</td></tr>
<tr><td>.*</td><td>arithm|calcul</td><td>arithmetic processing</td></tr>
<tr><td>.*</td><td>happy|angry</td><td>emotion perception</td></tr>
<tr><td>.*</td><td>rewarded</td><td>reward processing</td></tr>
<tr><td>.*</td><td>negative reward|punish|fail</td><td>loss processing</td></tr>
<tr><td>.*</td><td>succes[s]ful stop</td><td>response inhibition</td></tr>
<tr><td>.*</td><td>uncertain</td><td>decision under uncertainty</td></tr>
<tr><td>.*</td><td>checkerboard</td><td>visual perception,visual orientation</td></tr>
<tr><td>4337</td><td>FACES</td><td>feature comparison, response selection, respons...</td></tr>
<tr><td>4337</td><td>SHAPES</td><td>feature comparison, response selection, respons...</td></tr>
<tr><td>4337</td><td>PUNISH</td><td>response selection, response execution, punishm...</td></tr>
<tr><td>4337</td><td>PUNISH</td><td>response selection, response execution, reward ...</td></tr>
<tr><td>4337</td><td>MATH</td><td>response selection, response execution, auditor...</td></tr>
<tr><td>4337</td><td>STORY</td><td>response selection, response execution, auditor...</td></tr>
<tr><td>4337</td><td>CUE</td><td>response selection, cueing, updating, visual pe...</td></tr>
<tr><td>4337</td><td>LF</td><td>response execution, left toe response execution</td></tr>
<tr><td>4337</td><td>LH</td><td>response execution, left hand response execution</td></tr>
<tr><td>4337</td><td>RF</td><td>response execution, right toe response execution</td></tr>
<tr><td>4337</td><td>RH</td><td>response execution, right hand response execution</td></tr>
<tr><td>4337</td><td>MATCH</td><td>feature comparison, response selection, respons...</td></tr>
<tr><td>4337</td><td>REL</td><td>feature comparison, response selection, respons...</td></tr>
<tr><td>4337</td><td>RANDOM</td><td>response selection, motion detection</td></tr>
<tr><td>4337</td><td>TOM</td><td>response selection, motion detection, animacy p...</td></tr>
<tr><td>4337</td><td>0BK_BODY</td><td>response execution, working memory, body mainte...</td></tr>
<tr><td>4337</td><td>0BK_FACE</td><td>response execution, working memory, face mainte...</td></tr>
<tr><td>4337</td><td>0BK_PLACE</td><td>response execution, working memory, place maint...</td></tr>
<tr><td>4337</td><td>0BK_TOOL</td><td>response execution, working memory, tool mainte...</td></tr>
<tr><td>4337</td><td>2BK_BODY</td><td>response execution, working memory, body mainte...</td></tr>
<tr><td>4337</td><td>2BK_FACE</td><td>response execution, working memory, face mainte...</td></tr>
<tr><td>4337</td><td>2BK_PLACE</td><td>response execution, working memory, place maint...</td></tr>
<tr><td>4337</td><td>2BK_TOOL</td><td>response execution, working memory, tool mainte...</td></tr>
<tr><td>4343</td><td>stopsignal_GO</td><td>response execution, visual perception</td></tr>
<tr><td>4343</td><td>STOP_RIGHT|STOP_LEFT</td><td>response inhibition, visual perception, audition</td></tr>
<tr><td>4343</td><td>stopsignal_BLANK</td><td>visual perception</td></tr>
</table>

4343	bart_CONTROL_ACCEPT	response selection, decision, visual perception
1952	control condition of the pump action	response selection, decision, visual perception
4343	bart_CONTROL_CASHOUT	response selection, decision, visual perception
4343	bart_BALOON_ACCEPT	response selection, decision, decision under un...
1952	pump condition	response selection, decision, decision under un...
4343	bart_BALOON_CASHOUT	response selection, decision, decision under un...
1952	Response to acceptance	response selection, decision, decision under un...
4343	bart_BALOON_EXPLODE	loss processing, visual perception
1952	explosion	loss processing, visual perception
4343	pamenc_TASK	visual perception, visual word recognition, vis...
4343	pamenc_CONTROL	visual perception, visual recognition, visual w...
4343	pamret_CORRECT	visual perception, visual recognition, working ...
4343	pamret_INCORRECT	visual perception, visual recognition, working ...
4343	pamret_CONTROL	visual perception, visual word recognition, vis...
4343	scap_CORRECT	visual perception, visual localization, visual ...
4343	scap_INCORRECT	visual perception, visual localization, visual ...
4343	scap_NO_RESPONSE	visual perception, visual localization, visual ...
4343	taskswitch_SWITCH	visual perception, response execution, task swi...
4343	taskswitch_NOSWITCH	visual perception, response execution
4342	audonly	audition, right finger response execution
4342	vidonly	visual perception, right finger response execution
4342	audvid	audition, right finger response execution
4339	expression_control	visual perception, face perception
4339	expression_intention	visual perception, face perception, emotion rec...
4339	expression_sex	visual perception, face perception, gender disc...
4339	face_control	visual perception, face perception, face percep...
4339	face_sex	visual perception, face perception, gender disc...
4339	face_trusty	visual perception, face perception, facial trus...
4339	audio	audition
4339	calculaudio	audition, arithmetic processing
4339	calculvideo	visual perception, arithmetic processing
4339	clicDaudio	audition, response selection, response executio...
4339	clicDvideo	visual perception, response selection, response...
4339	clicGaudio	audition, response selection, response executio...
4339	clicGvideo	visual perception, response selection, response...
4339	computation	arithmetic processing
4339	damier_H	visual perception
4339	damier_V	visual perception
4339	object_grasp	motor control, motor planning, response selecti...
4339	object_orientation	visual perception, visual orientation
4339	rotation_hand	response selection, visual body recognition, me...
4339	rotation_side	response selection, visual body recognition
4339	saccade	visual perception, visual localization
4339	motor-cognitive	response selection, response execution, left fi...
4339	false_belief_audio	audition, auditory sentence recognition, senten...
4339	false_belief_video	visual perception, reading, visual word recogni...
4339	mecanistic_audio	audition, auditory sentence recognition, story ...
4339	mecanistic_video	visual perception, visual word recognition, sen...
4339	non_speech	audition
4339	speech	audition, voice perception, speech perception
4339	triangle_intention	response selection, motion detection, animacy p...
4339	triangle_random	response selection, motion detection

4341	left_auditory_click	audition, response selection, response executio...
4341	right_auditory_click	audition, response selection, response executio...
4341	left_visual_click	visual perception, response selection, response...
4341	right_visual_click	visual perception, response selection, response...
4341	checkerboard	visual perception,visual orientation
4341	visual_calculation	visual perception, arithmetic processing
4341	auditory_calculation	audition, arithmetic processing
4341	auditory&visual_calculation	visual perception, audition, arithmetic processing
4341	visual_sentences	visual perception, visual word recognition, sen...
4341	auditory_sentences	audition, auditory sentence recognition, sente...
4341	auditory&visual_sentences	visual perception, audition, visual word recogn...
2447	Left Hand	left hand response execution
2447	Left Leg	left toe response execution
2447	Tongue	tongue response execution
2447	dot motion coherence judgement	visual perception, visual localization
2447	noun/verb judgement	visual perception, visual word recognition
503	5720|5800|7000|7006|7010|7040|7060|7090|7100|71...	visual perception
503	2053|3051|3102|3120|3350|3500|3550|6831|9040|90...	negative emotion, visual perception
1964	3	negative emotion, visual perception
1964	4	negative emotion, visual perception
1964	5	negative emotion, visual perception

A.3.3 Cognitive Atlas relationships limitations.

Relationships between Cognitive Atlas concepts appear too incomplete to be used. Cognitive Atlas includes some of structure between its concepts, described in a rich graph database: concepts can have *kind of* or *part of* relationships. It also lists tasks designed to identify specific concepts. Yet, those relationships seem incomplete. Many concepts do not have any relationship and some obvious relations seem to be missing. For example, *auditory sentence comprehension* is not related to any other auditory concept and does not appear as being tested by the common *language processing fMRI task paradigm* whereas it should be.

A.4 Encoding method

Concept encoding with noise reduction. For a dataset $\{\mathbf{X}, \mathbf{Y}\}$, as a complement to our decoding goal of inferring the concepts $\widehat{\mathbf{y}}$ for any activation map over brain components $\mathbf{x}$, we compute the encoding map $\widehat{\mathbf{x}^l}$ for any concept l.

This is usually done by fitting a generalized linear model β (GLM) such that $\widehat{\mathbf{X}} = \mathbf{Y}\beta + \varepsilon$, where ε is the noise. Since the concept space is highly structured (for example, in the data used this work, $\mathbf{Y}^{perception} \approx \mathbf{Y}^{audition} + \mathbf{Y}^{visual\ perception}$), the design matrix $\widehat{\mathbf{Y}}$ can be ill-conditioned.

To better condition the matrix, we took inspiration from principal component regression (PCR,[44]). A regular PCR would mix the concept of interest design vector with all the others in the principal components of the design matrix. To avoid that, we fitted a GLM for each concept and use as regressors the original design vector for the concept of interest combined with the principal components for the design matrix of all the other concepts.

A.5 Decoding performance of NNoD variants

We compare the performance for different models in this setting in Table 4. In this setting, the explored models have quite similar results. Still, the non-linear binary logistic model (a 2-layers perceptron) achieves a slightly better average AUC across concepts. This model is trained to minimize a binary logistic loss $\mathscr{L}_{bin}$ with an elastic net (L1 + L2) regularization on the weights of both layers as in Eq (5), while applying dropout on the input and hidden layers.

Supplementary Table 1. Synonymy rules. Additional labels associated to initial vocabulary.

label	associated labels
analogy	analogical reasoning
association learning	association,learning
auditory arithmetic processing	arithmetic processing,audition
auditory perception	audition
autobiographical recall	autobiographical memory
emotional face recognition	emotion recognition
encoding	memory acquisition
facial recognition	face recognition
imagery	mental imagery
impulsivity	inhibition
intention	planning
interference resolution	interference control
language processing	language
listening	audition
logic	logical reasoning
motor learning	motor planning
narrative	story comprehension
prospective planning	planning
recall	memory retrieval
retrieval	memory retrieval
semantic knowledge	semantic processing
semantic information	semantic processing
sound perception	audition
visual face recognition	face recognition
visual imagery	mental imagery
visual representation	mental imagery

Supplementary Table 4. NNoD performance on the test set — 37 original concepts. WR@10 stands for Weighted Recall at 10.

Loss	Non-linear layers	AUC	WR@10
Binary Logistic	0	0.80	0.42
Binary Logistic	1	**0.81**	0.36
Binary Logistic	3	0.79	0.34
Multinomial Logistic	0	0.80	0.46
Multinomial Logistic	1	0.80	0.38
Multinomial Logistic	3	0.77	0.30

Training on the enriched dataset, as illustrated in Table 5, the explored architectures once again yield similar performance, with the non-linear binary logistic model yielding a slightly higher AUC.

A.6 Results on different data splits

We have performed additional experiments measuring the accuracy of terms identification on different studies. In Supplementary Figure 3 (top), results are shown using NeuroVault collections 1952 and 503. In Supplementary Figure 3 (bottom) results are shown using NeuroVault collections 504, 1964, 2447, 2606, 2978, 3235, 3467, 4022, 4339, 4341, 4815, 5802, 6298, 6299. The set of terms that can be tested varies with the test sets, but overall the accuracy is of the same order as with the IBC validation set.

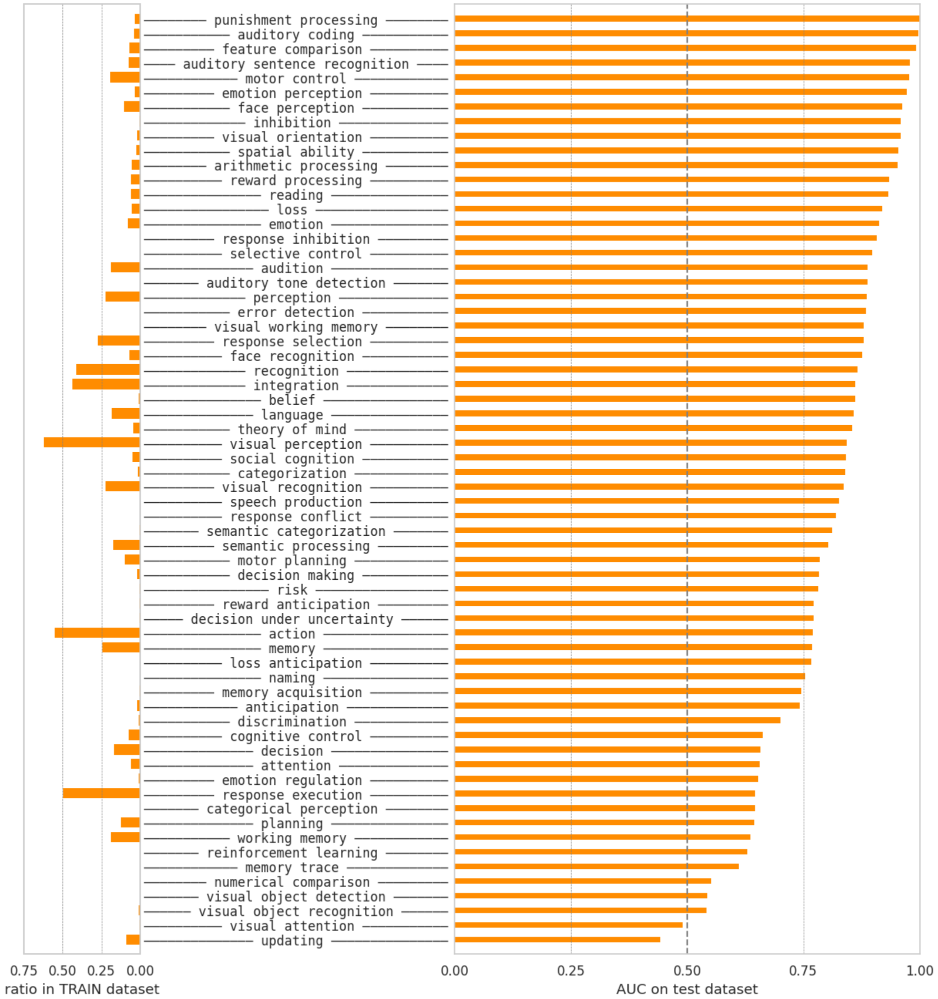

Supplementary Figure 3. Accuracy of the classifier on other validation folds.

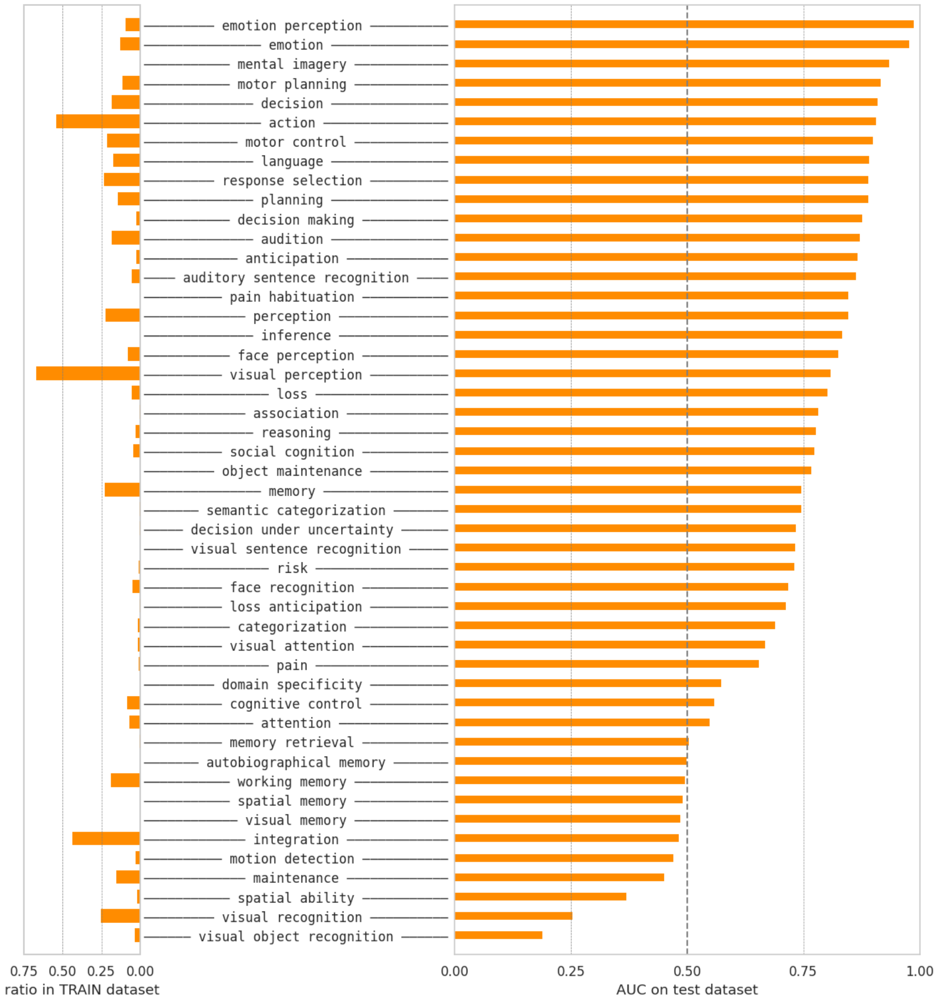

Supplementary Figure 3. Accuracy of the classifier on other validation folds (ctd). Here we obtain AUC scores using the same procedure as in the main text, but using different validation sets to measure performance. (top) score is obtained using NeuroVault collections 1952 and 503; (bottom) score is obtained using NeuroVault collections 504, 1964, 2447, 2606, 2978, 3235, 3467, 4022, 4339, 4341, 4815, 5802, 6298, 6299. The set of terms that can be tested varies with the test sets, but overall the accuracy is of the same order as with the IBC validation set.

Supplementary Table 5. NNoD performance on IBC — 51 enriched concepts.

Loss	Non-linear layers	AUC	WR@10
Binary Logistic	0	0.82	0.63
Binary Logistic	1	**0.84**	0.66
Binary Logistic	3	0.83	0.64
Multinomial Logistic	0	0.80	0.63
Multinomial Logistic	1	0.83	0.65
Multinomial Logistic	3	0.81	0.63

A.7 Encoding/Decoding maps

Supplementary Figure 4 displays encoding and decoding maps for 106 concepts. These maps illustrate the encoding models of all concepts learned over the training dataset (all NeuroVault except IBC) as well as the corresponding sensitivity analysis maps for the decoding models.

A.8 Vocabulary intersections with NeuroSynth and GCLDA

Here we provide the complete vocabulary lists corresponding to the intersections of IBC annotations, NNoD's vocabulary, and NeuroSynth and GCLDA's vocabularies.

expanded labels, intersection with NeuroSynth, 23 terms: action , arithmetic processing , attention , audition , decision , detection , discrimination , emotion , face perception , integration , language , loss , memory , motor control , punishment processing , response selection , reward processing , sentence processing , social cognition , theory of mind , updating , visual perception , working memory

expanded labels, intersection with GCLDA, 33 terms: action , arithmetic processing , attention , audition , auditory sentence recognition , decision , detection , discrimination , emotion , emotion perception , face perception , face recognition , facial trustworthiness recognition , integration , language , loss , maintenance , memory , motor control , pattern recognition , punishment processing , response execution , response selection , reward processing , semantic processing , sentence processing , social cognition , story comprehension , theory of mind , updating , visual perception , voice perception , working memory

exact labels, intersection with NeuroSynth, 14 terms: arithmetic processing , decision , detection , discrimination , emotion , memory , perception , punishment processing , recognition , response selection , reward processing , theory of mind , updating , working memory

exact labels, intersection with GCLDA, 20 terms: arithmetic processing , auditory sentence recognition , decision , detection , discrimination , emotion , face recognition , maintenance , memory , pattern recognition , perception , punishment processing , recognition , response execution , response selection , reward processing , story comprehension , theory of mind , updating , working memory

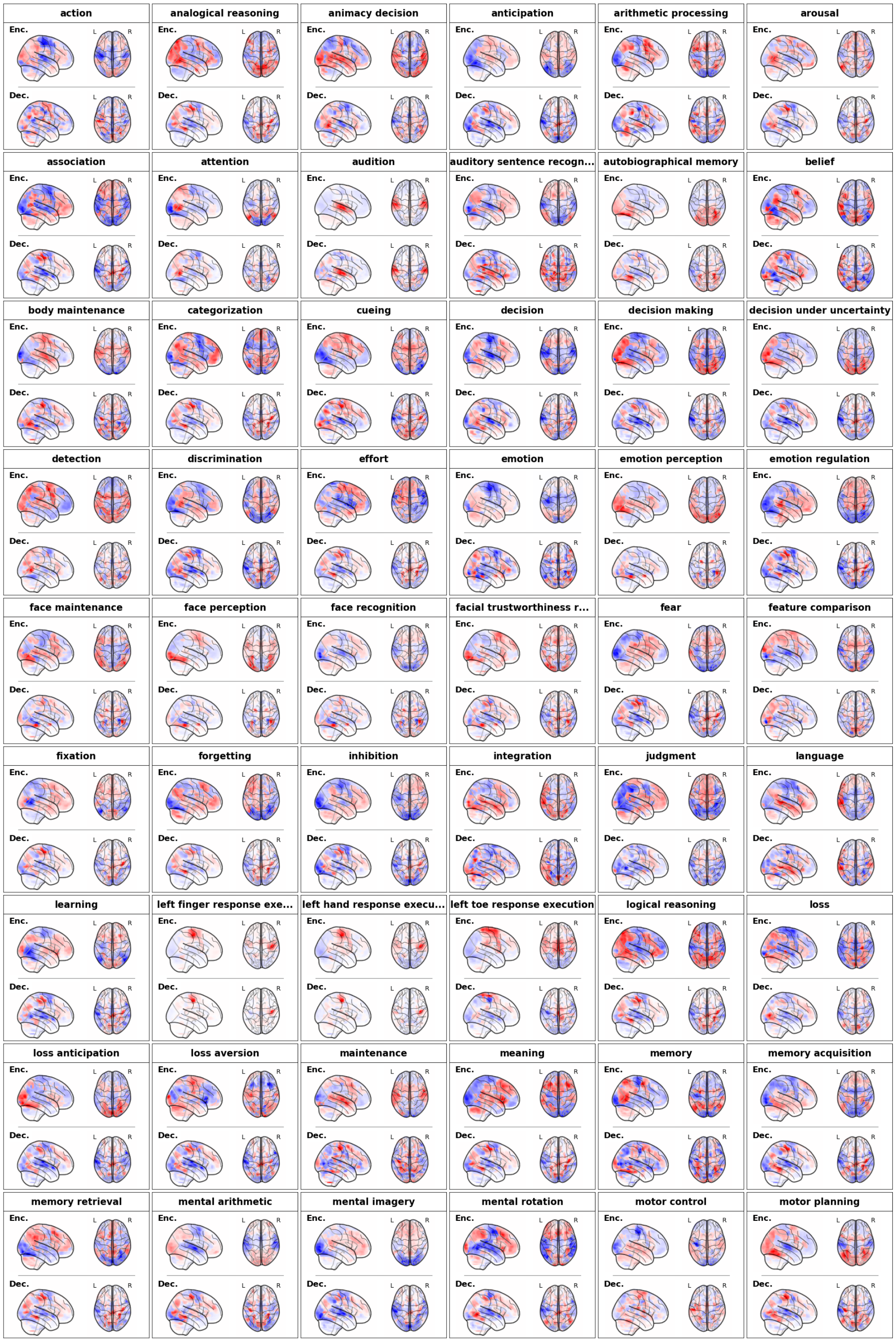

Supplementary Figure 4. Illustration of the encoding and decoding maps for 54 concepts learned from the enriched annotations.

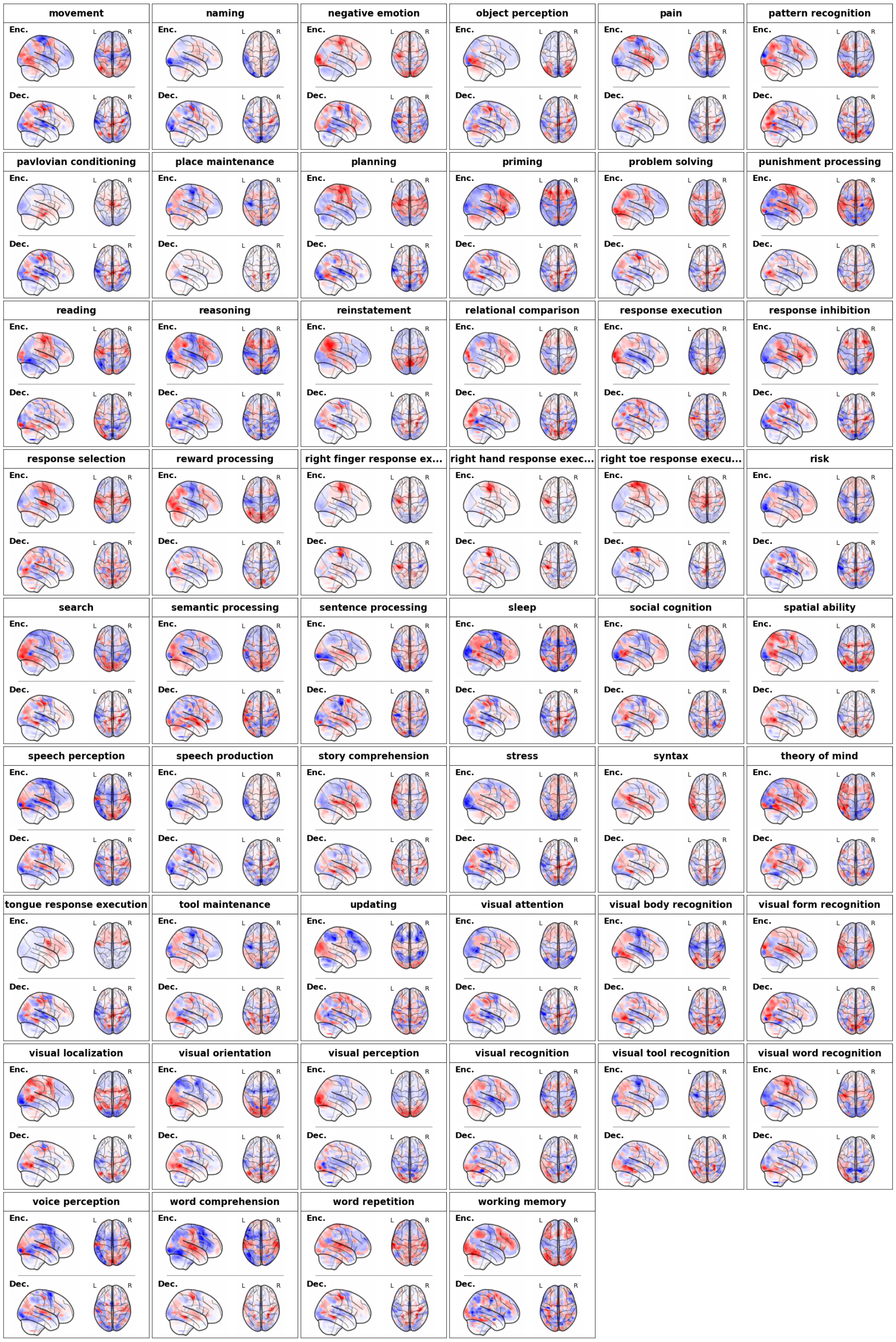

Supplementary Figure 4. Illustration of the encoding and decoding maps for 52 concepts learned from the enriched annotations.
